# A user-friendly and streamlined protocol for CRISPR/Cas9 genome editing in budding yeast

**DOI:** 10.1016/j.xpro.2022.101358

**Published:** 2022-06-10

**Authors:** Daniele Novarina, Andriana Koutsoumpa, Andreas Milias-Argeitis

**Affiliations:** 1 Molecular Systems Biology, Groningen Biomolecular Sciences and Biotechnology Institute, University of Groningen, Nijenborgh 4, 9747 AG Groningen, the Netherlands

**Keywords:** Genetics, Model Organisms, Molecular Biology, CRISPR

## Abstract

CRISPR/Cas9 technology allows accurate, marker-less genome editing. We report a detailed, robust, and streamlined protocol for CRISPR/Cas9 genome editing in *Saccharomyces cerevisiae*, based on the widely used MoClo-Yeast Toolkit (https://www.addgene.org/kits/moclo-ytk/). This step-by-step protocol guides the reader from sgRNA design to verification of the desired genome editing event and provides preassembled plasmids for cloning the sgRNA(s), making this technology easily accessible to any yeast research group.

For complete details on the use and execution of this protocol, please refer to Novarina et al. (2021).

## Highlights

• Complete and user-friendly protocol for CRISPR/Cas9 yeast genome editing
• sgRNA(s) and Cas9 cloning via Golden Gate Assembly with the MoClo-Yeast Toolkit
• Detailed description of repair fragment design for several genome editing applications
• Yeast one-step co-transformation with sgRNA(s)+Cas9 plasmid and repair fragment

## Abstract (editor highlights)

Publisher's note: Undertaking any experimental protocol requires adherence to local institutional guidelines for laboratory safety and ethics.

## Graphical abstract

**Figure:**
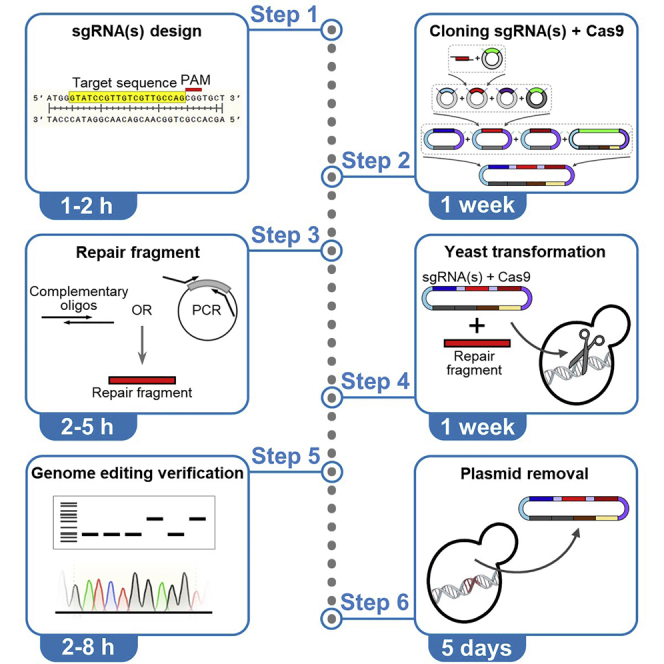

## Before you begin

**Figure 1:**
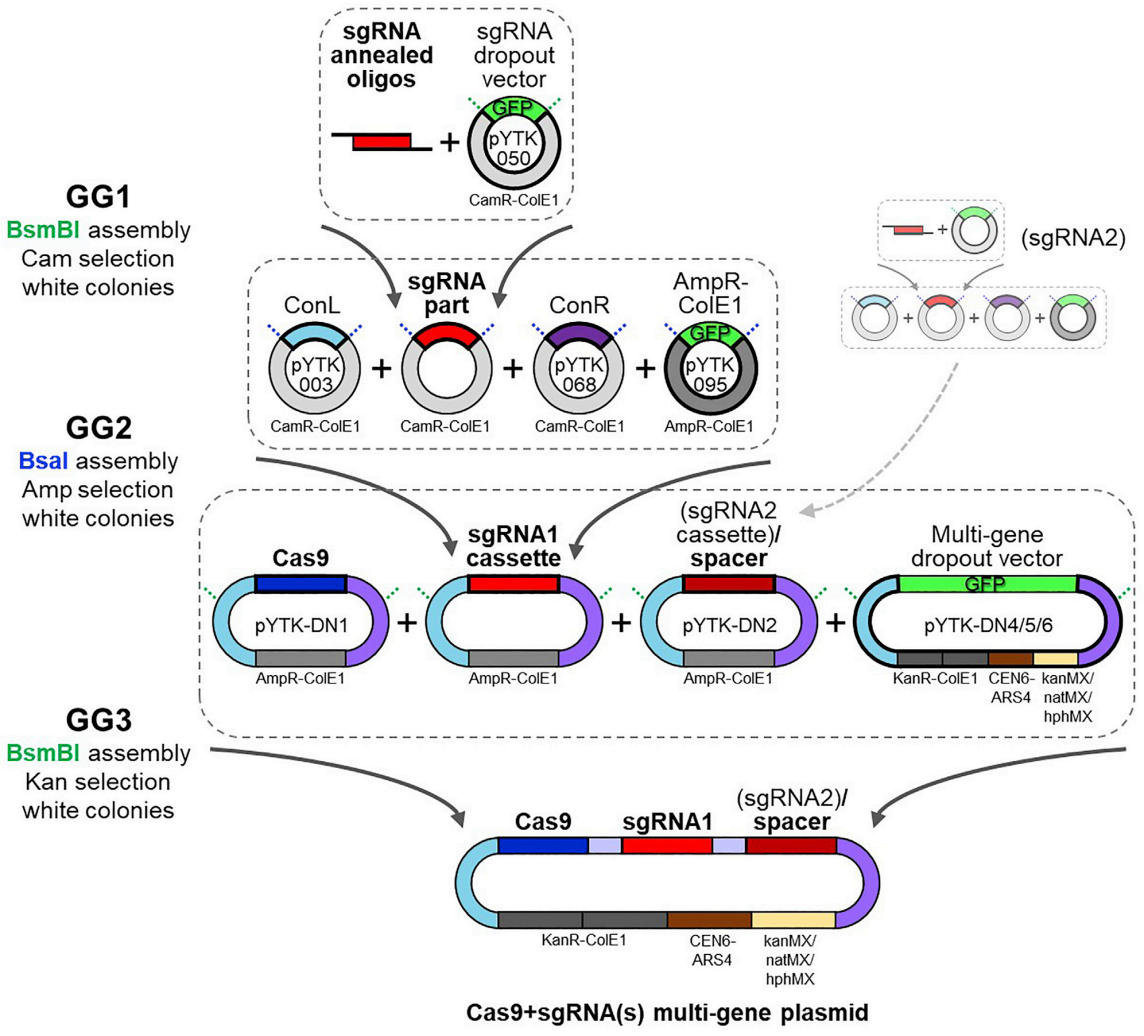
Schematic of the sgRNA(s) and Cas9 cloning through Golden Gate assembly The sgRNA and the Cas9 gene are cloned in a yeast expression vector through three consecutive Golden Gate assembly reactions. In the first Golden Gate assembly (GG1), partially overlapping annealed oligos containing the sgRNA sequence are cloned in the BsmBI-digested sgRNA dropout vector, yielding an sgRNA part plasmid (white colonies growing on chloramphenicol plates). In the second Golden Gate assembly (GG2), the sgRNA part is assembled with appropriate connectors in a second vector (AmpR-ColE1) after BsaI digestion, yielding an sgRNA cassette plasmid (white colonies growing on ampicillin plates). In the third Golden Gate assembly (GG3), the sgRNA cassette is assembled together with the Cas9 cassette and a spacer sequence (or a second sgRNA cassette) in a yeast expression vector (multi-gene dropout vector), yielding the Cas9+sgRNA(s) multi-gene plasmid (white colonies growing on kanamycin plates). Green dashed lines represent BsmBI restriction sites and blue dashed lines represent BsaI restriction sites.

This protocol describes a detailed procedure to perform CRISPR/Cas9 genome editing (Doudna and Charpentier, 2014) in *S. cerevisiae*, based on the MoClo-Yeast Toolkit (Lee et al., 2015) and a pre-existing protocol (Akhmetov et al., 2018). We provide detailed instructions for choosing the sgRNAs and designing partially overlapping complementary oligos for sgRNA cloning, as well as for the design and production of the repair fragments, depending on the nature of the desired genome editing event. One or two sgRNA are cloned in a yeast expression vector together with the Cas9 gene through three consecutive Golden Gate assembly reactions (Engler et al., 2008; Lee et al., 2015) (Figure 1). Co-transformation of the Cas9+sgRNA(s) multi-gene plasmid and the repair fragment(s) in yeast results in CRISPR genome editing. After verification of the genome editing event(s) by PCR and/or Sanger sequencing, the Cas9+sgRNA(s) multi-gene plasmid is removed from yeast cells. In our lab, we have successfully used this procedure to generate several mutations at the *SCH9* locus, encompassing full gene knockout, deletion of specific gene regions, and domain replacement (Novarina et al., 2021), as well as to perform gene knockouts and to introduce point mutations in several yeast genes (Guerra et al., 2021).

### Choose the sgRNA sequence

**Timing: 1–2 h**

**Figure 2:**
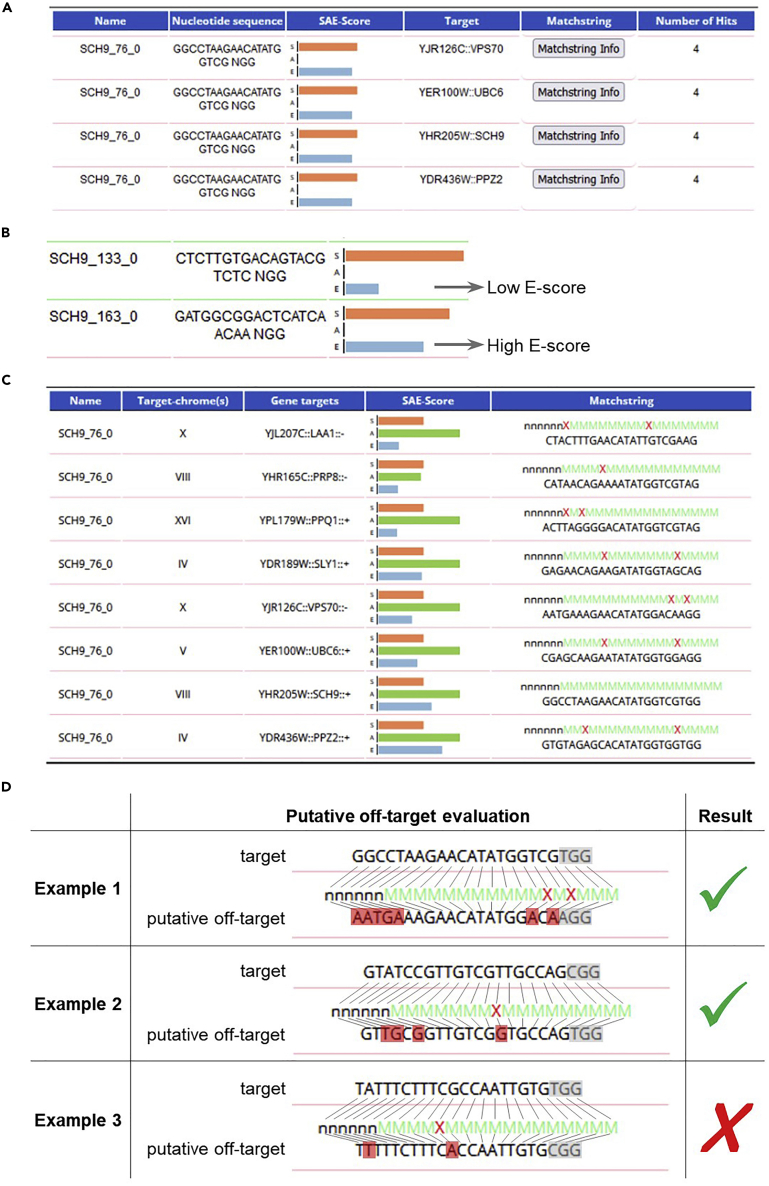
sgRNA design with E-CRISP (A) Output of sgRNA design. In the example, results for only one sgRNA (SCH9_76_0) are shown. (B) Example of two sgRNAs with different Efficacy score (E-score). (C) Output of sgRNA evaluation. In the example, the evaluation of the same sgRNA shown in Figure 2A is shown. In the Matchstring column, a green “M” represents a match, a red “X” represents a mismatch, and a black “n” represents a position not taken into account during the evaluation algorithm. Note that in the E-CRISP evaluation interface, the sequence of the putative off-target and the sequence representing the position of the mismatches are not aligned with respect to each other. In this example, the correct target (the *SCH9* gene) is shown, as well as 7 putative off-targets. Note that in the evaluation page more putative off-targets appear compared to the design page, since also the NAG sequence is permitted as PAM. Each putative off-target should be manually evaluated, taking into account the number and position of the mismatches with respect to the target sequence (see step 3 in section choose the sgRNA sequence for details). (D) Three examples of manual evaluation of putative off-targets. The PAM is highlighted in gray, while the mismatches are highlighted in red. In Example 1, two mismatches are located in close proximity to the PAM, and 5 other mismatches are located distal to the PAM: this is not an off-target, and can be ignored. In Example 2, one mismatch is located within the 10 PAM-proximal nucleotides, and three other mismatches are located distal to the PAM: this is also not an off-target, and can be ignored. In Example 3, one mismatch is located at the 10^th^ position distal to the PAM, and one other mismatch is located further distal to the PAM: since this could be an off-target in vivo, it is recommended to discard the corresponding sgRNA.

A potential sgRNA sequence for CRISPR/Cas9 editing of a desired genomic location is a 20 nucleotide sequence (target sequence), followed by the protospacer adjacent motif (PAM) NGG (where N can be any nucleotide). The target sequence determines the location, efficiency and specificity of Cas9 DNA cleavage for genome editing. Therefore, the choice of the proper target sequence is of great importance. We suggest the use of the E-CRISP (Heigwer et al., 2014) web application (http://www.e-crisp.org/) for identification and evaluation of putative sgRNA sequences (Figure 2).

***Note:*** The preferred PAM for *Streptococcus pyogenes* Cas9 (used in this protocol) is NGG, but targets with NAG PAM can also be cleaved, although less efficiently (Hsu et al., 2013; Jiang et al., 2013). Therefore, for the purpose of sgRNA design only NGG will be used, while for the detection of putative off-targets sequences with NAG PAM will also be taken into consideration.

1. Detect the possible sgRNA sequences in the E-CRISP “Design” section, using the parameters described below.
  a. Select organism: “Saccharomyces cerevisiae R64-1-1”.
  b. Select target region by gene symbol or sequence: insert the name of the target gene or paste a specific genomic sequence in FASTA format.
    i. For gene knockout insert the gene name or ORF symbol.
    ii. For N-terminal or C-terminal gene tagging, insert a sequence encompassing the 60 nucleotides upstream and the 60 nucleotides downstream the ATG starting codon (for N-terminal tagging) or the STOP codon (for C-terminal tagging).
    iii. For integration of a sequence at a specific genomic site, insert a 120-nucleotide sequence centered on the desired insertion site (or a longer sequence if the exact position of the integration site is not relevant).
    iv. For replacement of a genomic sequence, insert the sequence to be replaced.
    v. For point mutations, insert a ∼150 nucleotide sequence centered on the desired mutation site.
      ***Note:*** Increasing the length of the input sequence increases the chance of finding a good sgRNA sequence (i.e., with high cleavage efficiency and no off-targets). However, for some genome editing applications (such as N- and C-terminal tagging or introduction of point mutations) cleavage should occur in close proximity of the genome editing site, in order to ensure efficient editing. For this reason, we suggested different lengths of the input sequence for different genome editing applications.
  c. In the “Start application” section, select “medium”.
  d. Click on the “Display advanced options” to adjust the design parameters.
  e. In the “Design purpose” section adjust the parameters as follows to optimize the analysis for *S. cerevisiae* (if not specified, leave the default setting).
    i. 5′ preceding Base requirement: any (the default “G” comes from the cloning system used for human cells).
    ii. 3′ PAM: NGG.
    iii. exclude targets with poly T motif (it is a transcription termination signal for Pol III, that might hinder transcription of the sgRNA *in vivo*).
  f. In the “Gene annotation filtering” section deselect all options.
  g. In the “Off-target analysis” section set the following parameters (for all the genome editing applications listed in step 1b).
    i. Exclude designs with more than X off-targets: 20.
    ii. Number of 5′ mismatch positions: 6.
    iii. Tolerated edit distance to the target sequence: 2.
      ***Note:*** The recommended parameters in the “Off-target analysis” section are rather loose, allowing retrieval of sgRNAs with several potential off-targets. This is not a problem, because the user will evaluate the potential off-targets later, based on the position of the mismatches with respect to the PAM sequence (see step 3 of this section), which is not taken into account by the E-CRISP algorithm. Conversely, if the chosen parameters are too stringent here, the user will miss a lot of potential good sgRNA sequences.
      ***Optional:*** If you want to consider potential off-targets in the plasmid expressing Cas9 and the sgRNA, or other exogenous sequences already integrated in the target genome (i.e., fluorescent tags, markers, etc.), you can make use of the “Select to check for secondary off-targets” option.
  h. In the “Output” section adjust the parameters as follows.
    i. Maximum number of results per exon: select a high number (>50), otherwise some of the potential sgRNA sequences will not be displayed among the results.
    ii. Set the other parameters as you like; that will only influence the visualization of the results. Recommended settings:
       Create an image showing genomic context.
       Add TSS to the image.
       Add stop codons to the image.
       Add start codons to the image.
       Output the result table to the browser window.
       Produce additional information for the Matchstring.
  i. Press the “Start sgRNA search” button.
  j. An example of the output is displayed in Figure 2A.
2. Select a few candidate sgRNA sequences based on the following parameters:
  a. The position of the target sequence, graphically visualized at the bottom of the output page.
    i. For gene knockout the position is not relevant: the sgRNA can be located anywhere within the coding sequence.
    ii. For N-terminal tagging the sgRNA should be located across the ATG starting codon (optimal situation) or within a 120-nucleotides window centered on the ATG starting codon.
    iii. For C-terminal tagging the sgRNA should be located across the STOP codon (optimal situation) or within a 120-nucleotides window centered on the STOP codon.
    iv. For integration of an exogenous sequence at a desired genomic site, the sgRNA should be located within a 120-nucleotides window centered on the insertion site.
    v. For replacement of a genomic sequence, the sgRNA can be located anywhere within the sequence to be replaced (optimal), or within 60 bp outside of this sequence (upstream or downstream).
    vi. To introduce a point mutation at a desired genomic sequence, the sgRNA should be located across the mutation site (optimal situation) or within a 120-nucleotides window centered on the mutation site.
  b. The predicted cleavage efficiency, based on the Efficacy score (E-score) (Figure 2B): the higher the E-score, the higher the chance that the candidate sgRNA will work.
    ***Note:*** The Efficacy score (E-score) predicts Cas9-dependent cleavage efficiency for the given target sequence. However, there is no guarantee that a specific sgRNA works until direct *in vivo* validation. The Specificity score (S-score), calculated on the basis of the number of predicted off-targets should be ignored at this stage, because the potential off-targets will be later evaluated by the user. The Annotation score (A-score) can be ignored, since it is optimized on the human genome.
    ***Note:*** In the output window, the candidate sgRNAs are ranked according to the S-score, so do not hesitate to go down the list in search for good E-scores, even among sgRNA with several putative off-targets.

3. Evaluate the selected sgRNA sequences in the E-CRISP “Evaluation” section:
  a. Choose the following parameters in the “Select organism” section.
    i. Saccharomyces cerevisiae R64-1-1.
    ii. Number of 5′ mismatch positions ignored by the program: 6.
    iii. Tolerated edit distance to the target sequence: 2.
  b. In the “Enter target sequence” section, paste the 20-nucleotide target sequence (without the PAM) in FASTA format.
  c. Press the “Start” button.
    ***Note:*** The parameters chosen for the sgRNA evaluation are still very relaxed. With these settings the user will visualize all the putative “off-targets” with a more permissive PAM (NAG or NGG, as discussed above), with less than 3 mismatches in the 14 PAM-proximal bases and allowing any mismatch in the 6 PAM-distal bases (Figure 2C).
  d. Evaluate each of the putative “off-targets”, taking into account the number and the position of the mismatches with respect to the target sequence (Figure 2D), based on the following empirical rules:
    i. More than 3 mismatches in the target sequence abrogate cleavage by Cas9, based on studies on mammalian cells (Hsu et al., 2013).
    ii. 1 mismatch in the 10 PAM-proximal nucleotides is sufficient to strongly reduce cleavage by Cas9, based on yeast *in vivo* studies (Fu et al., 2016).
    iii. In summary, as a *safe rule for evaluation* of putative “off-targets”, if one potential “off-target” sequence has at least one mismatch in the 10 PAM-proximal nucleotides, and at least two extra mismatches in the whole 20-nucleotide sequence, you can be relatively certain that it will not be recognized by the selected sgRNA (i.e., it is not a real “off-target”).
      **CRITICAL:** While evaluating the total number of mismatches, you should also consider the sequence of the 6 most PAM-distal nucleotides.
      ***Note:*** By evaluating the putative “off-targets” as suggested (i.e., taking into account also the position of the mismatches with respect to the PAM), many of the putative “off-targets” can be neglected.
  e. Discard the sgRNA sequences that have off-targets that do not pass the *safe rule for evaluation* described above.
4. Select the two best sgRNA sequences (in terms of predicted efficiency and position relative to the editing site).

***Note:*** It is advised to design and clone in parallel multiple (at least 2) sgRNAs, since there is no guarantee that they will actually work *in vivo*. If several sgRNAs are cloned in parallel, in case the first tested sgRNA does not work, a second one will be immediately available for yeast transformation.

***Note:*** If you still have doubts that a putative “off-target” might be cleaved *in vivo*, you can sequence that specific genomic locus after performing the CRISPR genome editing, to verify that no unintended mutations have been introduced.

### Design oligos for sgRNA cloning

**Timing: 5 min**

Design partially overlapping complementary oligos that, after annealing, leave sticky ends compatible with BsmBI-digested pYTK050 (sgRNA dropout vector).

5. Copy the 20-nucleotide target sequence and use it to generate the oligos for sgRNA cloning as follows.
  a. Forward oligo: 5′-**GACTTT**- 20-nucleotides target sequence-3′.
  b. Reverse oligo: 5′-**AAAC**- reverse complement 20-nucleotides target sequence -**AA**-3′.
    **Example**: sgRNA sequence: 5′-GGCCTAAGAACATATGGTCGTGG-3′
          20-nt target sequence PAM
      Forward oligo: 5′-**GACTTT**GGCCTAAGAACATATGGTCG-3′
      Reverse oligo: 5′-**AAAC**CGACCATATGTTCTTAGGCC**AA**-3′
      Annealed oligos: 5′-**GACTTT**GGCCTAAGAACATATGGTCG-3′
          3′-**AA**CCGGATTCTTGTATACCAGC**CAAA**-5′
    ***Note:*** Please keep in mind the orientation of the 20-nucleotide target sequence while designing the oligos: the target sequence might be on the bottom strand.

### *In silico* Golden Gate assembly (optional)

**Timing: 1–3 h**

To generate maps of all plasmids obtained during the sgRNA and Cas9 cloning, and verify that the assemblies are done properly, it is recommended to perform all Golden Gate assemblies (see step-by-step method details) *in silico* beforehand, using the Benchling online tool (https://benchling.com/editor).

***Note:*** To use Benchling, a free account is needed.

***Note:*** An introductory tutorial for *in silico* Golden Gate Assembly with Benchling is available at the following link: https://help.benchling.com/en/articles/671283-clone-using-the-golden-gate-assembly-wizard.

6. The partially overlapping complementary oligos for sgRNA cloning are designed such that, after annealing, the resulting fragment harbors sticky ends compatible with BsmBI-digested pYTK050. However, the inputs for the *in silico* Golden Gate assembly are either circular plasmids or linear fragments with blunt ends, carrying the BsaI or BsmBI restriction sites. It is therefore necessary to create a Benchling-compatible version of the sgRNA by adding BsmBI sites at both ends, so that the result of *in silico* BsmBI digestion is identical to the fragment produced by annealing the partially overlapping complementary oligos designed in step 5 of the previous section.
  a. Take the sequence of the forward oligo for sgRNA cloning (see section design oligos for sgRNA cloning).
  b. Add the sequence *CGTCTCA* (containing the BsmBI restriction site) upstream the oligo sequence.
  c. Add the sequence *GTTTTGAGACG* (containing the BsmBI restriction site) downstream the oligo sequence.
  d. Paste the obtained sequence in a new file in SnapGene Viewer (or your favorite DNA sequence editing program) and save it in the desired folder.
    **Example**: Forward oligo: 5′-**GACTTT**GGCCTAAGAACATATGGTCG-3′
      Extended sequence for Benchling (added sequences in *Italics*):
       5′-*CGTCTCA***GACTTT**GGCCTAAGAACATATGGTCG*GTTTTGAGACG*-3′
      Double-stranded sequence for Benchling (BsmBI sites are highlighted in yellow):
       5′-*A***GACTTT**GGCCTAAGAACATATGGTCG*GTTTT*-3′
       3′-*T***CTGAAA**CCGGATTCTTGTATACCAGC*CAAAA*-5′
      Fragment after BsmBI digestion: 5′-**GACTTT**GGCCTAAGAACATATGGTCG-3′
            3′-**AA**CCGGATTCTTGTATACCAGC**CAAA**-5′

7. Open Benchling and create a new Project.
  a. Press the “Create” button (the “+“ symbol on the left).
  b. Choose “Project”.
  c. Name the project.
  d. Press “Create Project”.
8. Import all source sequences in Benchling.
  a. Press the “Create” button (the “+“ symbol on the left).
  b. Choose “DNA sequence”.
  c. Choose “Import DNA Sequences”.
  d. Import sequences of the desired plasmids via drag and drop or by uploading files form a folder.
9. Open the first plasmid of the Golden Gate Assembly in Benchling by clicking on it.
10. Create a new assembly file.
  a. Press the “Assembly Wizard” command in the bottom right corner.
  b. Select “Create New Assembly”.
  c. Select “Golden Gate” and press “Start”.
  d. Insert the name of the new plasmid (in the bottom right corner).
  e. Press the “Enzyme / Primer settings” button (under the new plasmid name).
  f. Select the appropriate Type IIS Enzyme (BsaI or BsmBI).
11. Select the fragments to be assembled.
  a. Select “Backbone” or “Insert” as appropriate.
  b. Select the appropriate fragment by choosing the coordinates in the “Set fragment” section (the plasmid map on the right helps identifying the fragment coordinates.
  c. Press “Set Fragment”.
  d. Open the next plasmid and repeat the three sub-steps above.
  e. To insert a new fragment in the assembly (if needed), select the “+” symbol in the bottom right corner.
  f. Insert all the fragments of the assembly in the proper order. Benchling will verify if the fragment edges are compatible for assembly.
12. Perform *in silico* assembly.
  a. When all fragments have been inserted, click “Assemble” in the bottom right corner.
  b. Select the Benchling folder where you want the new plasmid to be saved.
  c. Press “Select”.
13. When all fragments have been inserted, click “Assemble” in the bottom right corner.
14. Export the assembled sequences.
  a. Select the “account user” sign in the bottom left corner and chose “Data Export”.
  b. Select “Sequences GenBank Files”.
  c. Select the Benchling folder(s) where assembled sequences are located.
  d. Press the “Export” button.
  e. You will receive via email a link to the folder containing the files.

***Note:*** If you need to perform several assemblies with many parts in common, you can chose the option “Assembly” → ”Re-open” from the top right corner, and simply switch the parts you want to change.

### Preparation of *E. coli* competent cells

**Timing: 2–3 days**

15. Prepare and freeze down at −80°C DH5α *E. coli* competent cells using one of these recommended methods:
  a. Inoue method (Green and Sambrook, 2020).
  b. Calcium chloride method (Sambrook and Russell, 2006).

***Note:*** The Inoue method is more laborious, but yields competent cells with a higher transformation efficiency.

### Plasmid preparation

**Timing: 1 h**

16. Grow an overnight culture (12–20 h at 37°C) of *E. coli* strains pYTK050, pYTK003, pYTK068, pYTK095, pYTK-DN1, pYTK-DN2, pYTK-DN4, pYTK-DN5, pYTK-DN6 (and, if needed, also pYTK004, pYTK072, pYTK-DN3) in 2 mL LB medium containing the appropriate antibiotic for plasmid selection.
17. Extract the plasmid with a Miniprep kit according to the manufacturer’s instructions.
18. Measure plasmid concentration with the NanoDrop.
19. Store the plasmids at −20°C.

### Plates and media preparation

**Timing: 2–6 h**

20. Prepare the following plates for *E. coli* using standard lab recipes (Cold Spring Harbor Protocols, 2009):
  a. LB-chloramphenicol (25 μg/mL) agar plates.
  b. LB-ampicillin (100 μg/mL) agar plates.
  c. LB-kanamycin (50 μg/mL) agar plates.
21. Prepare the following LB liquid medium for *E. coli* using standard lab recipes (Cold Spring Harbor Protocols, 2016):
  a. LB-chloramphenicol (25 μg/mL).
  b. LB-ampicillin (100 μg/mL).
  c. LB-kanamycin (50 μg/mL).
22. Prepare YPD liquid medium for yeast using standard lab recipes (Cold Spring Harbor Protocols, 2017a).
23. Prepare YPD-agar plates, containing the one of the following drugs, depending on the resistance marker chosen for the Cas9+sgRNA(s) multi-gene plasmid (Cold Spring Harbor Protocols, 2017b).
  a. G418 (200 μg/mL) for the KanR marker (pYTK-DN4 vector).
  b. clonNAT (100 μg/mL) for the NatR marker (pYTK-DN5 vector).
  c. hygromycin B (200 μg/mL) for the HygR marker (pYTK-DN6 vector).

## Key resources table

**Table:** 

REAGENT or RESOURCE	SOURCE	IDENTIFIER
**Bacterial and virus strains**		

*Escherichia coli*: DH5α	New England Biolabs	Cat#C2988J

**Chemicals, peptides, and recombinant proteins**		

Polyethylene glycol (PEG)	Sigma-Aldrich	Cat#202444
Carrier ssDNA (Deoxyribonucleic acid sodium salt from salmon testes)	Sigma-Aldrich	Cat#D1626
Chloramphenicol	Sigma-Aldrich	Cat#C0378
Ampicillin	Duchefa Biochemie	Cat#A0104
Kanamycin sulfate	Sigma-Aldrich	Cat#60615
G418 disulfate salt	Sigma-Aldrich	Cat#A1720
clonNAT	Jena Bioscience	Cat#AB-102
Hygromycin B	Duchefa Biochemie	Cat#H0192
YPD Broth powder	Formedium	Cat#CCM0210
LB Broth Miller powder	Formedium	Cat#LMM0102
Yeast extract powder	Formedium	Cat#YEA02
Peptone	Formedium	Cat#PEP02
Glucose (D-(+)-Glucose monohydrate)	Sigma-Aldrich	Cat#49159
Agar	Formedium	Cat#AGR10
Bacto Tryptone	Thermo Fisher Scientific	Cat#211705
NaCl	Sigma-Aldrich	Cat#S9888
Lithium acetate dihydrate	Sigma-Aldrich	Cat#62393
Sodium dodecyl sulfate (SDS)	Sigma-Aldrich	Cat#L3771

**Critical commercial assays**		

Miniprep kit	MACHEREY-NAGEL	Cat#740588
PCR purification kit	MACHEREY-NAGEL	Cat#740609
T4 DNA Ligase Reaction Buffer	New England Biolabs	Cat#B0202S
T7 DNA Ligase	New England Biolabs	Cat#M0318S
BsmBI	New England Biolabs	Cat#R0580S
BsaI	New England Biolabs	Cat#R0535S
EcoRI-HF	New England Biolabs	Cat#R3101S
Q5 High-Fidelity DNA Polymerase	New England Biolabs	Cat#M0491S
5× Q5 reaction buffer	New England Biolabs	Cat#B9027S
10× Buffer rCutSmart	New England Biolabs	Cat#B6004S
PvuII-HF	New England Biolabs	Cat#R3151S

**Experimental models: Organisms/strains**		

*Saccharomyces cerevisiae:* YSBN6 (*MAT***a***ho::HphMX4*)	(Canelas et al., 2010)	N/A

**Oligonucleotides**		

Primer sgRNA-seq-fwd: CGAGGAGCCGTAATTTTTGC	This paper	N/A

**Recombinant DNA**		

pYTK003 (selection: chloramphenicol)	(Lee et al., 2015)	Addgene Plasmid #65110
pYTK004 (selection: chloramphenicol)	(Lee et al., 2015)	Addgene Plasmid #65111
pYTK050 (selection: chloramphenicol)	(Lee et al., 2015)	Addgene Plasmid #65157
pYTK068 (selection: chloramphenicol)	(Lee et al., 2015)	Addgene Plasmid #65175
pYTK072 (selection: chloramphenicol)	(Lee et al., 2015)	Addgene Plasmid #65179
pYTK095 (selection: ampicillin)	(Lee et al., 2015)	Addgene Plasmid #65202
pYTK-DN1 (selection: ampicillin)	This paper	Addgene Plasmid #180282
pYTK-DN2 (selection: ampicillin)	This paper	Addgene Plasmid #180283
pYTK-DN3 (selection: ampicillin)	This paper	Addgene Plasmid #180284
pYTK-DN4 (selection: kanamycin)	This paper	Addgene Plasmid #180285
pYTK-DN5 (selection: kanamycin)	This paper	Addgene Plasmid #180286
pYTK-DN6 (selection: kanamycin)	This paper	Addgene Plasmid #180287

**Software and algorithms**		

E-CRISP	(Heigwer et al., 2014)	http://www.e-crisp.org/
SnapGene Viewer	SnapGene	https://www.snapgene.com/snapgene-viewer/
Benchling	Benchling	https://benchling.com/editor
CRISPR-Cas9 toolbox MoClo Assembly Excel spreadsheet	This paper	Data S1

**Other**		

NanoDrop 2000 Spectrophotometer	Thermo Scientific	Cat#ND-2000
T100 Thermal Cycler	Bio-Rad	Cat#186-1096

## Materials and equipment

**Table:** YPD liquid medium

Reagent	Final concentration	Amount
YPD Broth powder	50 g/L	50 g
ddH_2_O	n/a	1 L
**Total**	**n/a**	**1 L**

Filter-sterilize. YPD liquid medium can be stored at room temperature (20°C–25°C) for (at least) 6 months.

**Table:** YPD agar plates (with antibiotics)

Reagent	Final concentration	Amount
Yeast extract	1% (w/v)	10 g
Peptone	2% (w/v)	20 g
Agar	2% (w/v)	20 g
ddH_2_O	n/a	up to 920 mL
Autoclave		
25% (w/v) Glucose	2% (w/v)	80 mL
200 mg/mL G418 (optional)	200 μg/mL	1 mL
100 mg/mL clonNAT (optional)	100 μg/mL	1 mL
200 mg/mL HygB (optional)	200 μg/mL	1 mL
**Total**	**n/a**	**1 L**

Adjust ddH_2_O before autoclaving. Add (filter-sterilized) glucose and antibiotics when the medium has cooled down to ∼65°C. YPD agar plates (with or without antibiotics) can be stored at 4°C for (at least) 3 months, provided that they are placed in a plastic bag to prevent them from drying out.

**Table:** LB liquid medium (with antibiotics)

Reagent	Final concentration	Amount
LB Broth Miller powder	25 g/L	25 g
100 mg/mL ampicillin (optional)	100 μg/mL	1 mL
25 mg/mL chloramphenicol (optional)	25 μg/mL	1 mL
50 mg/mL kanamycin (optional)	50 μg/mL	1 mL
ddH_2_O	n/a	1 L
**Total**	**n/a**	**1 L**

Autoclave or filter-sterilize. If the medium is autoclaved, add antibiotics when the medium has cooled down to ∼65°C. It is also possible to add antibiotics just before use. LB liquid medium without antibiotics can be stored at room temperature (20°C–25°C) for (at least) 6 months. LB liquid medium with antibiotics can be stored at 4°C for (at least) 3 months.

**Table:** LB agar plates (with antibiotics)

Reagent	Final concentration	Amount
Yeast extract	0.5% (w/v)	5 g
Tryptone	1% (w/v)	10 g
NaCl	1% (w/v)	10 g
Agar	1.5% (w/v)	15 g
ddH_2_O	n/a	up to 1 L
Autoclave		
100 mg/mL ampicillin (optional)	100 μg/mL	1 mL
25 mg/mL chloramphenicol (optional)	25 μg/mL	1 mL
50 mg/mL kanamycin (optional)	50 μg/mL	1 mL
**Total**	**n/a**	**1 L**

Adjust ddH_2_O before autoclaving. Add antibiotics when the medium has cooled down to ∼65°C.

LB agar plates (with antibiotics) can be stored at 4°C for (at least) 3 months, provided that they are placed in a plastic bag to prevent them from drying out.

***Alternatives:*** Instead of preparing *E. coli* competent cells, it is also possible to buy competent cells, for instance NEB 5-alpha Competent *E. coli* (High Efficiency) (New England Biolabs, C2987).

***Alternatives:*** We have optimized this protocol using the S288C-derived prototrophic YSBN6 yeast genetic background (Canelas et al., 2010), but any *S. cerevisiae* laboratory strain can be used.

***Alternatives:*** Instead of the NanoDrop, any other equivalent spectrophotometer can be used for nucleic acid quantification.

***Alternatives:*** Instead of E-CRISP, the CRISPOR online tool (http://crispor.org) could be used for sgRNA design and off-target evaluation, with the limitation that input sequences longer than 2,300 nucleotides are not allowed.

***Alternatives:*** For Golden Gate Assembly and PCR, any thermocycler can be used.

## Step-by-step method details

### *In vitro* oligos annealing for sgRNA cloning

**Timing: 1 h**

The partially overlapping complementary oligos are annealed *in vitro* to obtain a double-stranded Golden Gate-compatible fragment (ds-sgRNA) for cloning into the sgRNA dropout vector (pYTK050).

1. Prepare a 100 μM dilution of the oligos in ddH_2_O.
2. Mix 10 μL oligo forward and 10 μL oligo reverse in a PCR tube.
3. Anneal the oligos in a thermocycler using the following program:

**Table:** 

*In vitro* annealing conditions		
Steps	Temperature	Time
Denaturation	95°C	5 min
Annealing	55°C	15 min
Annealing	25°C	15 min
Hold	4°C	forever

### First Golden Gate assembly (GG1): sgRNA part

**Timing: 2.5 days**

The sgRNA is cloned in the sgRNA dropout vector (pYTK050) to obtain the sgRNA part plasmid (Figure 1).

**Figure 3:**
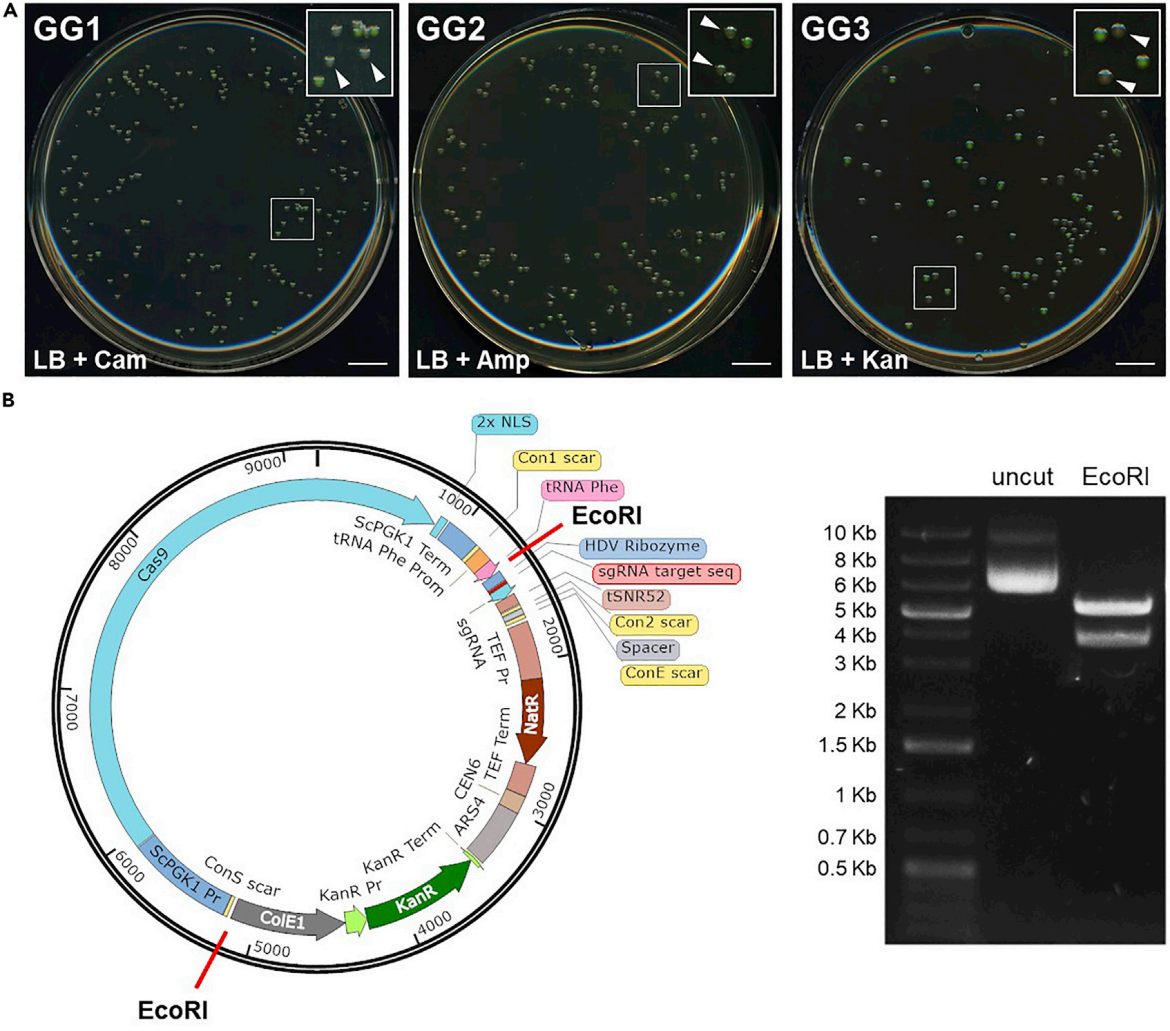
Examples of Golden Gate assembly (A) Examples of plates from *E. coli* transformation after Golden Gate assembly (steps 4–15). For each plate, in the insert at the top right corner white arrowheads indicate two white colonies that could be selected for the next steps. Scale bar, 1 cm. (B) Example of plasmid verification via restriction digestion (step 16). The Cas9+sgRNA multi-gene plasmid map created with Benchling and annotated with SnapGene is shown on the left. Red lines mark the two EcoRI restriction sites. The result of DNA gel electrophoresis of the uncut and EcoRI-digested plasmid is shown on the right. The tested plasmid displays the expected restriction digestion pattern (5.4 Kb + 3.8 Kb).

**CRITICAL:** The vectors used in all three Golden Gate assembly steps (pYTK050, pYTK095, pYTK-DN4, pYTK-DN5 and pYTK-DN6) contain a *GFP* gene driven by a promoter for expression in *E. coli*. This allows visual detection of *E. coli* colonies transformed with the undigested vector, since these colonies appear green on the plate. Conversely, correct Golden Gate assembly results in loss of the *GFP* gene, yielding white *E. coli* colonies (Figure 3A).

4. Set up the Golden Gate reaction.
  a. Prepare a 1:500 dilution of the ds-sgRNA fragment obtained in step 3.
  b. Prepare the GG1 Golden mix in a PCR tube:

**Table:** 

Component	Amount
ds-sgRNA (diluted 1:500)	0.4 μL
pYTK050 (sgRNA dropout plasmid)	20 fmol
10× T4 buffer	1 μL
T7 ligase	0.5 μL
BsmBI	0.5 μL
ddH_2_O	up to 10 μL
**Total**	**10 μL**

**CRITICAL:** T7 ligase enzyme is used in combination with T4 ligase buffer, it is not a typo.

***Note:*** You can use the Excel sheet “GG1 sgRNA” from the “CRISPR-Cas9 toolbox MoClo Assembly spreadsheet” (Data S1) to calculate the μL DNA of pYTK050.

5. Perform the Golden Gate reaction in a thermocycler:

**Table:** 

Golden gate cycling conditions			
Steps	Temperature	Time	Cycles
Digestion	42°C	2 min	25 cycles
Ligation	16°C	5 min	
Final digestion	60°C	10 min	1
Heat inactivation	80°C	10 min	1
Hold	4°C	forever

**Pause point:** Golden Gate products can be stored at 4°C for a few days.

6. Transform 3 μL of the Golden Gate Assembly product (sgRNA part) in 25 μL Inoue competent *E. coli* cells (or 5 μL sgRNA part in 50 μL CaCl_2_ competent cells).
  a. Thaw competent cells on ice.
  b. Add 3–5 μL of the Golden Gate Assembly product obtained in step 5 (sgRNA part) to the competent cells. Mix gently by pipetting up and down or by flicking the tube 4–5 times. Do not vortex.
  c. Place the mixture on ice for 30 min.
  d. Heat shock at 42°C for 30 s.
  e. Place tubes in ice for 2 min.
  f. Add 950 μL of room-temperature (20°C–25°C) LB liquid medium to the tube.
  g. Incubate the tube at 37°C for 60 min, shaking vigorously (250 rpm).
  h. Plate 100 μL of the cell suspension on a LB + chloramphenicol plate.
  i. Incubate the plate overnight (12–20 h) at 37°C.

***Note:*** On the transformation plate a mixture of white and green colonies will grow. Green colonies contain the undigested pYTK050 (expressing the GFP gene), while white colonies contain a plasmid that has lost the GFP gene as a consequence of the Golden Gate Assembly (Figures 1 and 3A).

***Note:*** A PCR-based screening of the colonies is not needed, since the white/green colonies visual screen allows exclusion of the colonies containing the non-digested vector.

**Pause point:** plates can be stored at 4°C for a several days after transformation.

7. Prepare the sgRNA part plasmid. Troubleshooting 1.
  a. Inoculate 1 or 2 white colonies in 3 mL LB + chloramphenicol liquid medium. Troubleshooting 2.
  b. Grow overnight (12–20 h) at 37°C.
  c. Use 2 mL of the overnight culture to extract the sgRNA part plasmid with a Miniprep kit.
  d. Measure plasmid concentration with the NanoDrop.
  e. Use the remaining 1 mL for glycerol stock.

***Optional:*** Test 1 or 2 plasmids by restriction digestion. If the 20-nucleotide target sequence contains a restriction site, digest with the corresponding enzyme, otherwise you can digest with PvuII (cuts once in the CamR gene).

**Table:** 

Component	Amount
DNA	X μL (∼400 ng)
10× Buffer rCutSmart	2 μL
PvuII-HF	0.4 μL
ddH_2_O	up to 20 μL
**Total**	**20 μL**

Incubate for 15 min at 37°C. Add 4 μL of 6× loading dye and load 20 μL on agarose gel. Load also 1 μL of undigested plasmid for comparison.

***Note:*** The Golden Gate reaction works virtually always, so it is not needed to test each plasmid by restriction digestion. We suggest to test only the plasmid obtained through the last Golden Gate Assembly reaction (steps 12–16). Keep all the *E. coli* plates at 4°C until the end of the whole cloning procedure, so that, if accidentally something went wrong, you can go back to the appropriate step and use another colony to prepare plasmid DNA.

**Pause point:** The sgRNA part plasmid can be stored at −20°C for several months.

### Second Golden Gate assembly (GG2): sgRNA cassette

**Timing: 2.5 days**

The sgRNA is assembled in a transcriptional unit, to obtain the sgRNA cassette plasmid. Based on the connectors used, the sgRNA can be assembled as a TU2 or TU3 transcriptional unit (Figure 1).

8. Set up the Golden Gate reaction.
  a. For cloning the TU2 sgRNA cassette plasmid, prepare the GG2 Golden mix in a PCR tube as follows:

**Table:** 

Component	Amount
pYTK003 (ConL1)	20 fmol
sgRNA part plasmid	20 fmol
pYTK068 (ConR2)	20 fmol
pYTK095 (AmpR-ColE1)	20 fmol
10× T4 buffer	1 μL
T7 ligase	0.5 μL
BsaI	0.5 μL
ddH_2_O	up to 10 μL
**Total**	**10 μL**
  b. For cloning the TU3 sgRNA cassette plasmid, prepare the GG2 Golden mix in a PCR tube as follows:

**Table:** 

Component	Amount
pYTK004 (ConL2)	20 fmol
sgRNA part plasmid	20 fmol
pYTK072 (ConRE)	20 fmol
pYTK095 (AmpR-ColE1)	20 fmol
10× T4 buffer	1 μL
T7 ligase	0.5 μL
BsaI	0.5 μL
ddH_2_O	up to 10 μL
**Total**	**10 μL**

    **CRITICAL:** T7 ligase enzyme is used in combination with T4 ligase buffer, it is not a typo.
    ***Note:*** You can use the Excel sheets “GG2 sgRNA1” and “GG2 sgRNA2” from the “CRISPR-Cas9 toolbox MoClo Assembly spreadsheet” to calculate the μL DNA of every plasmid for assembly of TU2 and TU3 sgRNA cassette plasmids, respectively.

9. Perform the Golden Gate reaction in the thermocycler, using the same cycling conditions described in step 5.

**Pause point:** Golden Gate products can be stored at 4°C for a few days.

10. Transform 3 μL of the Golden Gate Assembly product (sgRNA cassette plasmid) in 25 μL Inoue competent *E. coli* cells (or 5 μL sgRNA part in 50 μL CaCl_2_ competent cells) as described in step 6, with the following difference:
  a. Plate 100 μL of the cell suspension on a LB + ampicillin plate.

***Note:*** On the transformation plate a mixture of white and green colonies will grow. Green colonies contain the undigested pYTK095 (expressing the GFP gene), while white colonies contain a plasmid that has lost the GFP gene as a consequence of the Golden Gate Assembly (Figures 1 and 3A).

**Pause point:** plates can be stored at 4°C for a several days after transformation.

11. Prepare the sgRNA cassette plasmid. Troubleshooting 1.
  a. Inoculate 1 or 2 white colonies in 3 mL LB + ampicillin liquid medium. Troubleshooting 2.
  b. Grow overnight (12–20 h) at 37°C.
  c. Use 2 mL of the overnight culture to extract the sgRNA part plasmid with a Miniprep kit.
  d. Measure plasmid concentration with the NanoDrop.
  e. Use the remaining 1 mL for glycerol stock.

***Optional:*** Test 1 or 2 plasmids by restriction digestion. If the 20-nucleotide target sequence contains a restriction site, digest again with the corresponding enzyme, otherwise you can digest with XbaI (cuts once just upstream the ConL connector).

**Table:** 

Component	Amount
DNA	X μL (∼400 ng)
10× Buffer rCutSmart	2 μL
XbaI	0.4 μL
ddH_2_O	up to 20 μL
**Total**	**20 μL**

Incubate for 15 min at 37°C. Add 4 μL of 6× loading dye and load 20 μL on agarose gel. Load also 1 μL of undigested plasmid for comparison.

**Pause point:** The sgRNA cassette plasmid can be stored at −20°C for several months.

### Third Golden Gate assembly (GG3): Cas9+sgRNA(s) multi-gene plasmid

**Timing: 2.5 days**

The Cas9 cassette (pYTK-DN1) and the gRNA cassette(s) are cloned in a yeast expression vector (pYTK-DN4, pYTK-DN5 or pYTK-DN6) to obtain the multi-gene plasmid (Figure 1).

12. Set up the Golden Gate reaction.
  a. For cloning the Cas9+sgRNA1(TU2) into the yeast expression vector, prepare the GG3 Golden mix in a PCR tube as follows:

**Table:** 

Component	Amount
pYTK-DN1 (TU1 Cas9 cassette)	20 fmol
sgRNA1 cassette plasmid (TU2)	20 fmol
pYTK-DN2 (TU3 spacer cassette)	20 fmol
yeast vector∗	20 fmol
10× T4 buffer	1 μL
T7 ligase	0.5 μL
BsmBI	0.5 μL
ddH_2_O	up to 10 μL
**Total**	**10 μL**

∗ pYTK-DN4 (KanR vector) or pYTK-DN5 (NatR vector) or pYTK-DN6 (HygR vector).
  b. For cloning the Cas9+sgRNA2(TU3) into the yeast expression vector, prepare the GG3 Golden mix in a PCR tube as follows:

**Table:** 

Component	Amount
pYTK-DN1 (TU1 Cas9 cassette)	20 fmol
pYTK-DN3 (TU2 spacer cassette)	20 fmol
sgRNA2 cassette plasmid (TU3)	20 fmol
yeast vector∗	20 fmol
10× T4 buffer	1 μL
T7 ligase	0.5 μL
BsmBI	0.5 μL
ddH_2_O	up to 10 μL
**Total**	**10 μL**

∗ pYTK-DN4 (KanR vector) or pYTK-DN5 (NatR vector) or pYTK-DN6 (HygR vector).
  c. For cloning the Cas9+sgRNA1(TU2)+sgRNA2(TU3) into the yeast expression vector, prepare the GG3 Golden mix in a PCR tube as follows:

**Table:** 

Component	Amount
pYTK-DN1 (TU1 Cas9 cassette)	20 fmol
sgRNA1 cassette plasmid (TU2)	20 fmol
sgRNA2 cassette plasmid (TU3)	20 fmol
yeast vector∗	20 fmol
10× T4 buffer	1 μL
T7 ligase	0.5 μL
BsmBI	0.5 μL
ddH_2_O	up to 10 μL
**Total**	**10 μL**

∗ pYTK-DN4 (KanR vector) or pYTK-DN5 (NatR vector) or pYTK-DN6 (HygR vector).

    **CRITICAL:** T7 ligase enzyme is used in combination with T4 ligase buffer, it is not a typo!
    ***Note:*** You can use the Excel sheets “GG3 Cas9-sgRNA1-spacer”, “GG3 Cas9-spacer-sgRNA2” and “GG3 Cas9-sgRNA1-sgRNA2” from the “CRISPR-Cas9 toolbox MoClo Assembly spreadsheet” to calculate the μL plasmid DNA for assembly of Cas9 and the gRNA cassette(s) into the yeast expression vector.

13. Perform the Golden Gate reaction in the thermocycler, using the same cycling conditions described in step 5.

**Pause point:** Golden Gate products can be stored at 4°C for a few days.

14. Transform 3 μL of the Golden Gate Assembly product (Cas9+sgRNA(s) multi-gene plasmid) in 25 μL Inoue competent *E. coli* cells (or 5 μL sgRNA part in 50 μL CaCl_2_ competent cells) as described in step 6, with the following difference:
  a. Plate 100 μL of the cell suspension on a LB + kanamycin plate.

***Note:*** On the transformation plate a mixture of white and green colonies will grow. Green colonies contain the undigested yeast vector (expressing the GFP gene), while white colonies contain a plasmid that has lost the GFP gene as a consequence of the Golden Gate Assembly (Figures 1 and 3A).

**Pause point:** plates can be stored at 4°C for a several days after transformation.

15. Prepare the Cas9+sgRNA(s) multi-gene plasmid. Troubleshooting 1.
  a. Inoculate 1 or 2 white colonies in 3 mL LB + kanamycin liquid medium. Troubleshooting 2.
  b. Grow overnight (12–20 h) at 37°C.
  c. Use 2 mL of the overnight culture to extract the sgRNA part plasmid with a Miniprep kit.
  d. Measure plasmid concentration with the NanoDrop.
  e. Use the remaining 1 mL for glycerol stock.
16. Test 1 or 2 plasmids by restriction digestion with EcoRI (Figure 3B), which cuts 2 times in the correctly assembled Cas9+sgRNA1(TU2) and Cas9+sgRNA2(TU3) multi-gene plasmids, and 3 times in the correctly assembled Cas9+sgRNA1(TU2)+sgRNA2(TU3) multi-gene plasmid (unless a third or fourth restriction site, respectively, is present in the 20-nucleotides target sequence).
  a. Prepare the digestion mix:

**Table:** 

Component	Amount
DNA	X μL (∼400 ng)
10× Buffer rCutSmart	2 μL
EcoRI-HF	0.4 μL
ddH_2_O	up to 20 μL
**Total**	**20 μL**
  b. Incubate 15 min at 37°C.
  c. Add 4 μL of 6× loading dye and load 20 μL on agarose gel. Load also 1 μL of undigested plasmid for comparison. Analyze fragments via DNA electrophoresis.
    ***Optional:*** Verify the sgRNA insert by sequencing with primer sgRNA-seq-fwd.
    **Pause point:** The Cas9+sgRNA(s) multi-gene plasmid can be stored at −20°C for several months.
    ***Note:*** The following sections (steps 17–31) contain detailed instructions for the design and production of the repair fragment for different genome editing applications (i.e., gene knockout, N- and C-terminal tagging, sequence integration at a specific genomic location, sequence replacement, and introduction of point mutations). Please choose the appropriate section based on the desired genome editing application, then proceed with yeast transformation (step 32).
    ***Note:*** In the following sections, we refer to the genomic sequence that is modified as “genome editing site” (can span from a single nucleotide to several kilobases, depending on the application), and to the genomic sequence bound and cleaved by Cas9 as “target sequence”. For some applications these two sequences (partially) overlap, as in the case of gene deletion or sequence integration. In other instances, they can be located several nucleotides apart, which can happen sometimes with point mutations or N- and C-terminal tagging.

### Design and production of the repair fragment for gene deletion

**Timing: 2 h**

**Figure 4:**
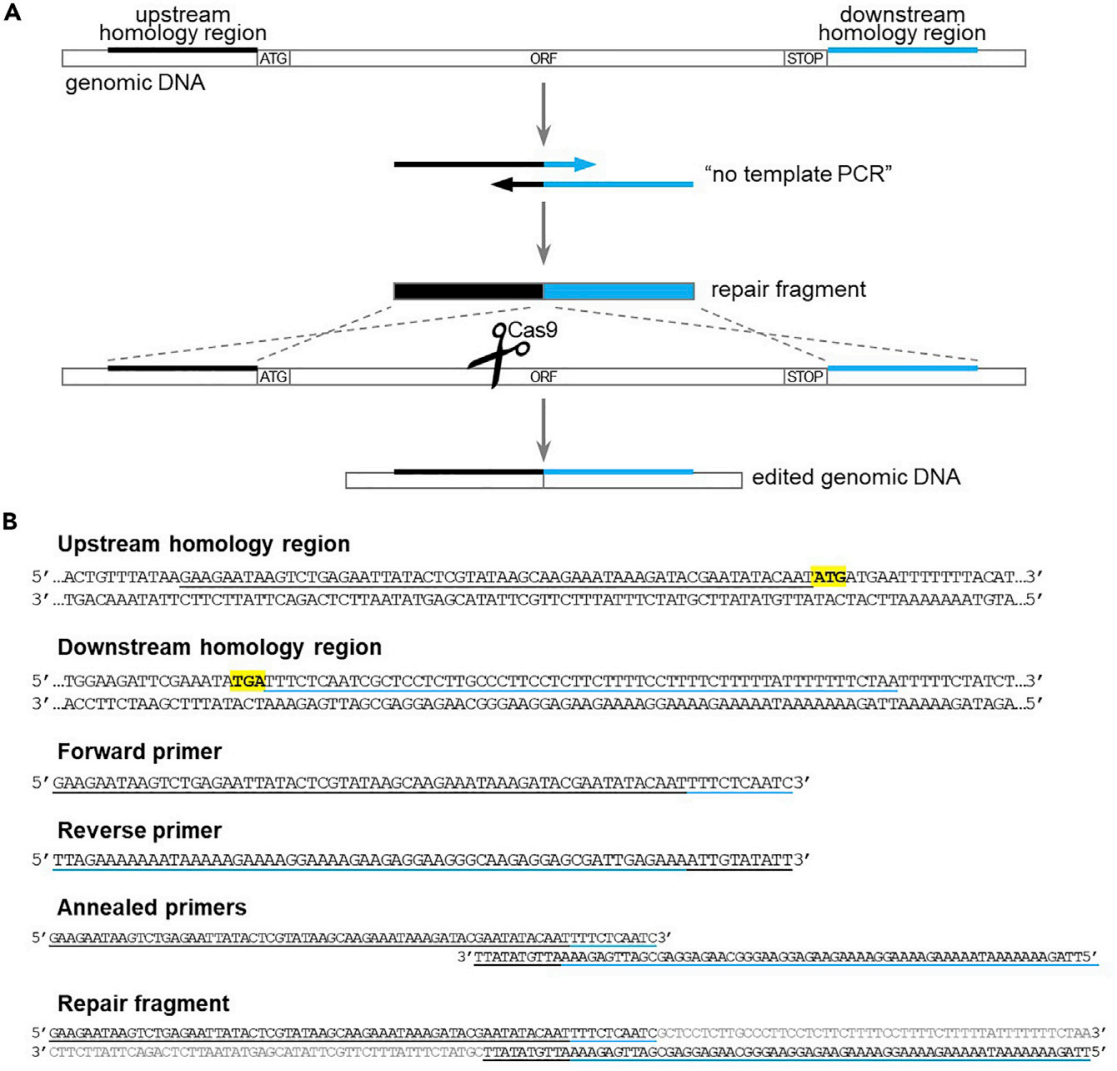
Design of repair fragment for gene deletion (A) Schematic of the repair fragment design. The repair fragment is obtained through “no template PCR” amplification (steps 17 and 18) of partially overlapping primers containing the homology regions upstream the ATG start codon (in black) and downstream the STOP codon (in blue). After Cas9-dependent cleavage within the ORF sequence, repair fragment integration results in complete deletion of the gene sequence. (B) Example of primers design for gene deletion (step 17). ATG start codon and STOP codon are highlighted in yellow. See main text for details.

The repair fragment for gene deletion is made up of the 60 nucleotides upstream the ATG start codon, followed by the 60 nucleotides downstream the STOP codon. The repair fragment is obtained by PCR amplification of partially overlapping primers (“no template PCR”, Figure 4).

***Note:*** Studies in yeast suggest that it might be possible to use a single-stranded oligonucleotide instead of a double-stranded DNA fragment as a repair template for gene deletion (Dicarlo et al., 2013; Storici et al., 2003), even though we have not tested it in our hands.

17. Design partially overlapping primers.
  a. Forward primer: 60 nucleotides upstream ATG + 10 nucleotides downstream STOP.
  b. Reverse primer: reverse complement 60 nucleotides downstream STOP + reverse complement 10 nucleotides upstream ATG.
18. Amplify the repair fragment via “no template PCR”. Troubleshooting 3.
  a. Prepare the PCR mix:

**Table:** 

Component	Amount
5× Q5 buffer	40 μL
10 mM dNTPs	4 μL
10 μM forward primer	10 μL
10 μM reverse primer	10 μL
Q5 Polymerase	2 μL
H_2_O (Milli-Q)	134 μL
**Total**	**200 μL**
  b. Divide the PCR mix in 4 PCR tubes (50 μL per tube).
  c. Perform the PCR reaction in the thermocycler with the following program:

**Table:** 

PCR cycling conditions			
Steps	Temperature	Time	Cycles
Initial Denaturation	98°C	30 s	1
Denaturation	98°C	10 s	20 cycles
Annealing	50°C∗	30 s	
Extension	72°C	20 s	
Denaturation	98°C	10 s	10 cycles
Annealing + extension	72°C	20 s	
Final extension	72°C	5 min	1
Hold	4°C	forever	

∗ Annealing temperature of the overlapping region between the 2 primers (20 nucleotides). To calculate the annealing temperature, you can use the NEB Tm calculator tool (https://tmcalculator.neb.com).

    ***Note:*** The PCR program performs 20 cycles with an annealing temperature corresponding to the overlapping region (20 nucleotides), and then another 10 cycles with an annealing temperature = extension temperature (=72°C), corresponding to annealing of the whole primers (70 nucleotides).
  d. Pool the 4 PCR reactions together.
    ***Optional:*** Load 5 μL PCR product on agarose gel and verify PCR via DNA electrophoresis (expected band size: 120 bp).

19. Proceed with yeast transformation (step 32).

### Design and production of the repair fragment for N-terminal tagging

**Timing: 2–5 h**

**Figure 5:**
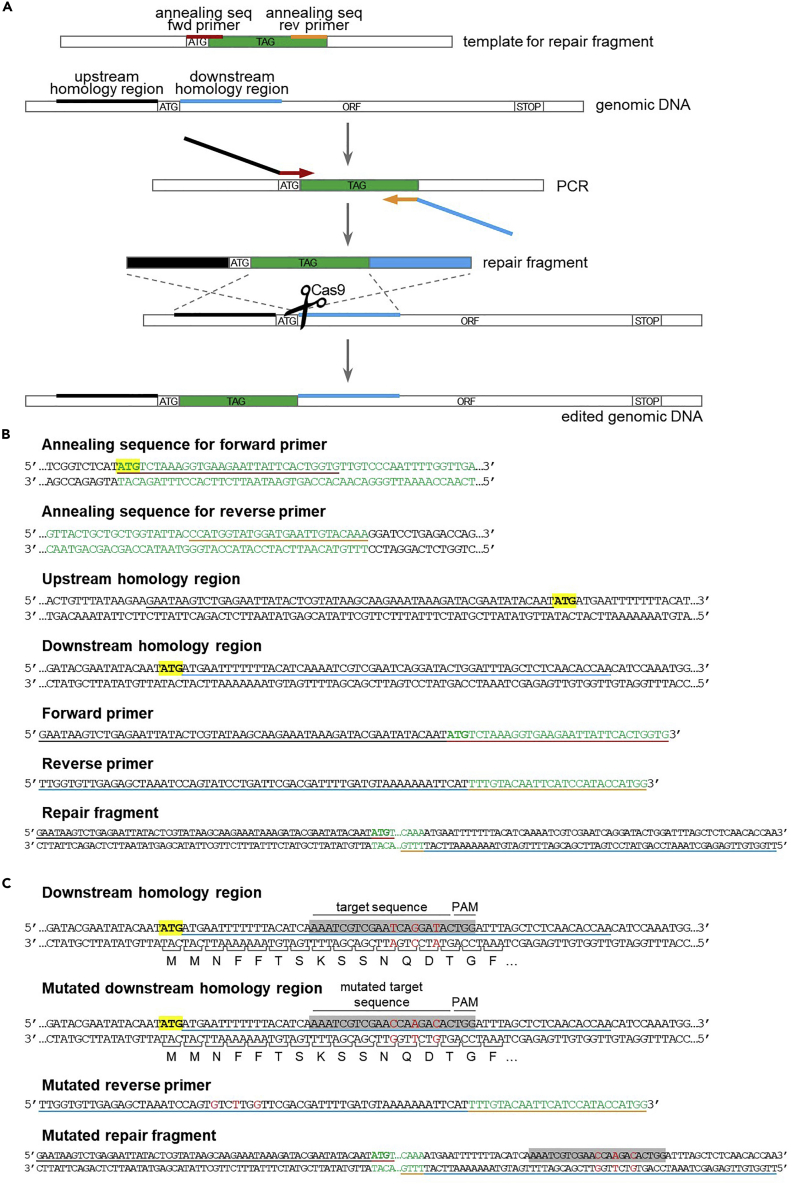
Design of repair fragment for N-terminal tagging (A) Schematic of the repair fragment design. The repair fragment is obtained through PCR amplification of the tag (in green) with primers annealing at the beginning (in dark red) and at the end (in orange) of the tag, and carrying tails for the homology regions upstream (in black) and downstream (in blue) the ATG start codon. After Cas9-dependent cleavage near the ATG start codon, repair fragment integration results in introduction of the tag at the N-terminal of the gene. (B) Example of primers design for N-terminal tagging (step 20). The ATG start codon is highlighted in yellow. (C) Introduction of synonymous mutations in the target sequence to prevent Cas9 cleavage after repair (step 21). The sgRNA sequence is highlighted in gray. The possible base changes for synonymous mutations are marked in red. The amino acid sequence is displayed using the one-letter code under the DNA sequence. See main text for details.

The repair fragment for N-terminal tagging of a gene is made up of the 57 nucleotides upstream the ATG start codon, followed by the tag, followed by the 60 nucleotides downstream the ATG start codon. The repair fragment is obtained by standard PCR amplification (Figure 5). If the sgRNA does not span through the ATG start codon, it is also necessary to introduce one or more (synonymous) mutations in the sgRNA sequence, to prevent re-cutting by Cas9 after repair, as described in detail below.

**CRITICAL:** As the wide majority of tags start with an ATG codon, this design yields a repair fragment with 60 nucleotides of homology at both ends (Figure 5). However, if the tag does not start with an ATG codon, it is necessary to add the ATG codon to the upstream homology region (step 20c).

20. In case the sgRNA sequence spans through the ATG start codon, design the primers for the repair fragment as follows.
  a. Design the annealing sequence for the forward primer on the top strand of the template for tag amplification, according to the following rules:
    i. Start at the beginning of the tag sequence (from the ATG) and extend the sequence in 5′ to 3′ direction.
    ii. The annealing sequence should be at least 20 nucleotides long.
    iii. If possible, the CG content should be between 35% and 65%.
    iv. The last nucleotide should be G or C, and the last 5 nucleotides should enclose at least 2 G/C.
    v. The Tm of the annealing sequence should be compatible with the Tm of the annealing sequence for the reverse primer (no more than 5°C difference).
  b. Design the annealing sequence for the reverse primer on the top strand of the template for tag amplification, according to the following rules:
    i. Start at the end of the tag sequence (excluding the STOP codon, in case it is present) and extend the sequence in 3′ to 5′ direction.
    ii. The annealing sequence should be at least 20 nucleotides long.
    iii. If possible, the CG content should be between 35% and 65%.
    iv. The last nucleotide should be G or C, and the last 5 nucleotides should enclose at least 2 G/C.
    v. The Tm of the annealing sequence should be compatible with the Tm of the annealing sequence for the forward primer (no more than 5°C difference).
  c. Design the homology regions for genomic integration.
    i. Upstream homology region: 57 nucleotides upstream the ATG starting codon.
    ii. Downstream homology region: 60 nucleotides downstream the ATG starting codon.
  d. Combine the annealing regions and the homology regions to create the primers.
    i. Forward primer: upstream homology region + annealing sequence for the forward primer.
    ii. Reverse primer: reverse complement downstream homology region + reverse complement annealing sequence for the reverse primer.
  e. Amplify the repair fragment through standard PCR.

***Note:*** If the sgRNA sequence spans through the ATG start codon, introduction of an N-terminal sequence will automatically disrupt the sgRNA sequence.

21. In case the sgRNA sequence does not span through the ATG start codon, design the primers for the repair fragment as follows.
  a. Design the primers as indicated in step 20.
  b. Introduce point mutations in the upstream homology region or in the downstream homology region of the primers, in order to disrupt the sgRNA sequence, but still preserving the amino acid sequence (if the sgRNA encompasses the coding region). This can be done in two ways:
    i. Mutate one of the “Gs” of the PAM site (NGG) so that the target sequence is no longer recognized (but avoid NAG, because it can still be recognized as a PAM).
    ii. Mutate at least 2 bases among the 10 PAM-proximal bases (this is enough to prevent binding of the sgRNA).
  c. Amplify the repair fragment through standard PCR.
22. Proceed with yeast transformation (step 32).

### Design and production of the repair fragment for C-terminal tagging

**Timing: 2–5 h**

**Figure 6:**
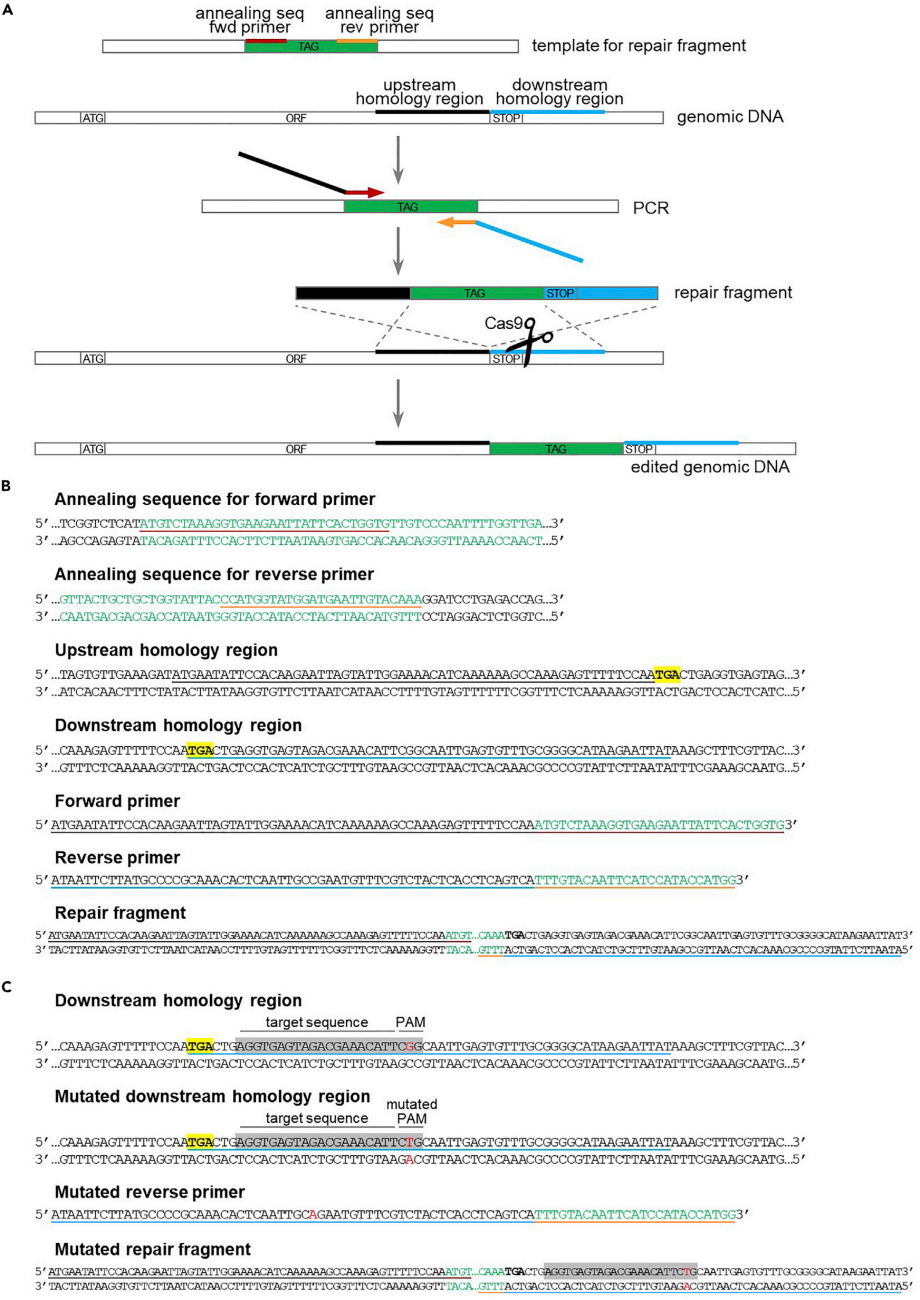
Design of repair fragment for C-terminal tagging (A) Schematic of the repair fragment design. The repair fragment is obtained through PCR amplification of the tag (in green) with primers annealing at the beginning (in dark red) and at the end (in orange) of the tag, and carrying tails for the homology regions upstream (in black) and downstream (in blue) the STOP codon. After Cas9-dependent cleavage near the STOP codon, repair fragment integration results in introduction of the tag at the C-terminal of the gene (before the STOP codon). (B) Example of primers design for C-terminal tagging (step 23). The STOP codon is highlighted in yellow. (C) Mutation of the PAM sequence to prevent Cas9 cleavage after repair (step 24). The sgRNA sequence is highlighted in gray. The mutated base is marked in red. See main text for details.

The repair fragment for C-terminal tagging of a gene is made up of the 60 nucleotides upstream the STOP codon, followed by the tag, followed by the STOP codon, followed by the 57 nucleotides downstream the STOP codon. The repair fragment is obtained by standard PCR amplification (Figure 6). If the sgRNA does not span through the STOP codon, it is also necessary to introduce one or more (synonymous) mutations in the sgRNA sequence, to prevent re-cutting by Cas9 after repair, as described in detail below.

23. In case the sgRNA sequence spans through the STOP codon, design the primers for the repair fragment as follows.
  a. Design the annealing sequence for the forward primer on the top strand of the template for tag amplification, according to the following rules:
    i. Start at the beginning of the tag sequence and extend the sequence in 5′ to 3′ direction.
    ii. The annealing sequence should be at least 20 nucleotides long.
    iii. If possible, the CG content should be between 35% and 65%.
    iv. The last nucleotide should be G or C, and the last 5 nucleotides should enclose at least 2 G/C.
    v. The Tm of the annealing sequence should be compatible with the Tm of the annealing sequence for the reverse primer (no more than 5°C difference).
  b. Design the annealing sequence for the reverse primer on the top strand of the template for tag amplification, according to the following rules:
    i. Start at the end of the tag sequence and extend the sequence in 3′ to 5′ direction.
    ii. The annealing sequence should be at least 20 nucleotides long.
    iii. If possible, the CG content should be between 35% and 65%.
    iv. The last nucleotide should be G or C, and the last 5 nucleotides should enclose at least 2 G/C.
    v. The Tm of the annealing sequence should be compatible with the Tm of the annealing sequence for the forward primer (no more than 5°C difference).
  c. Design the homology regions for genomic integration.
    i. Upstream homology region: 60 nucleotides upstream the STOP codon.
    ii. Downstream homology region: STOP codon + 57 nucleotides downstream the STOP codon.
  d. Combine the annealing regions and the homology regions to create the primers.
    i. Forward primer: upstream homology region + annealing sequence for the forward primer.
    ii. Reverse primer: reverse complement downstream homology region + reverse complement annealing sequence for the reverse primer.
  e. Amplify the repair fragment through standard PCR.

***Note:*** If the sgRNA sequence spans through the STOP codon, introduction of a C-terminal sequence will automatically disrupt the sgRNA sequence.

24. In case the sgRNA sequence does not span through the STOP codon, design the primers for the repair fragment as follows.
  a. Design the primers as indicated in step 23.
  b. Introduce point mutations in the upstream homology region or in the downstream homology region of the primers, in order to disrupt the sgRNA sequence, but still preserving the amino acid sequence (if the sgRNA encompasses the coding region). This can be done in two ways:
    i. Mutate one of the “Gs” of the PAM site (NGG) so that the target sequence is no longer recognized (but avoid NAG, because it can still be recognized as a PAM).
    ii. Mutate at least 2 bases among the 10 PAM-proximal bases (this is enough to prevent binding of the sgRNA).
  c. Amplify the repair fragment through standard PCR.
25. Proceed with yeast transformation (step 32).

### Design and production of the repair fragment for sequence integration or sequence replacement

**Timing: 2–5 h**

**Figure 7:**
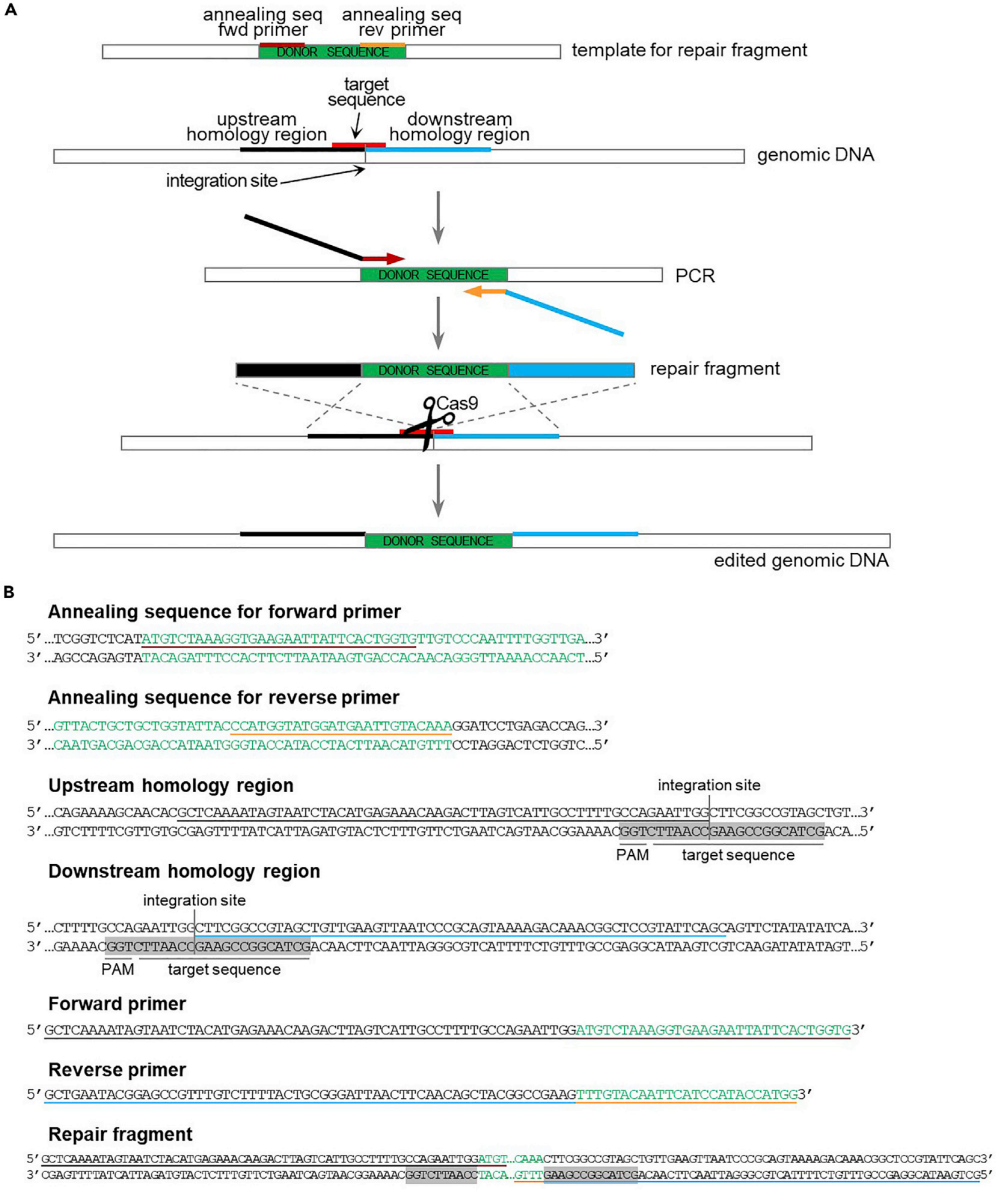
Design of repair fragment for sequence integration at a desired genomic location (A) Schematic of the repair fragment design. The integration site is located within the target sequence (marked in red). The repair fragment is obtained through PCR amplification of the donor sequence (in green) with primers annealing at the beginning (in dark red) and at the end (in orange) of the sequence, and carrying tails for the homology regions upstream (in black) and downstream (in blue) the integration site. After Cas9-dependent cleavage near the integration site, repair fragment integration results in the insertion of the donor sequence at the integration site with concomitant disruption of the target sequence. (B) Example of primers design for sequence integration (step 26). The sgRNA sequence (located on the bottom strand) is highlighted in gray. See main text for details.

**Figure 8:**
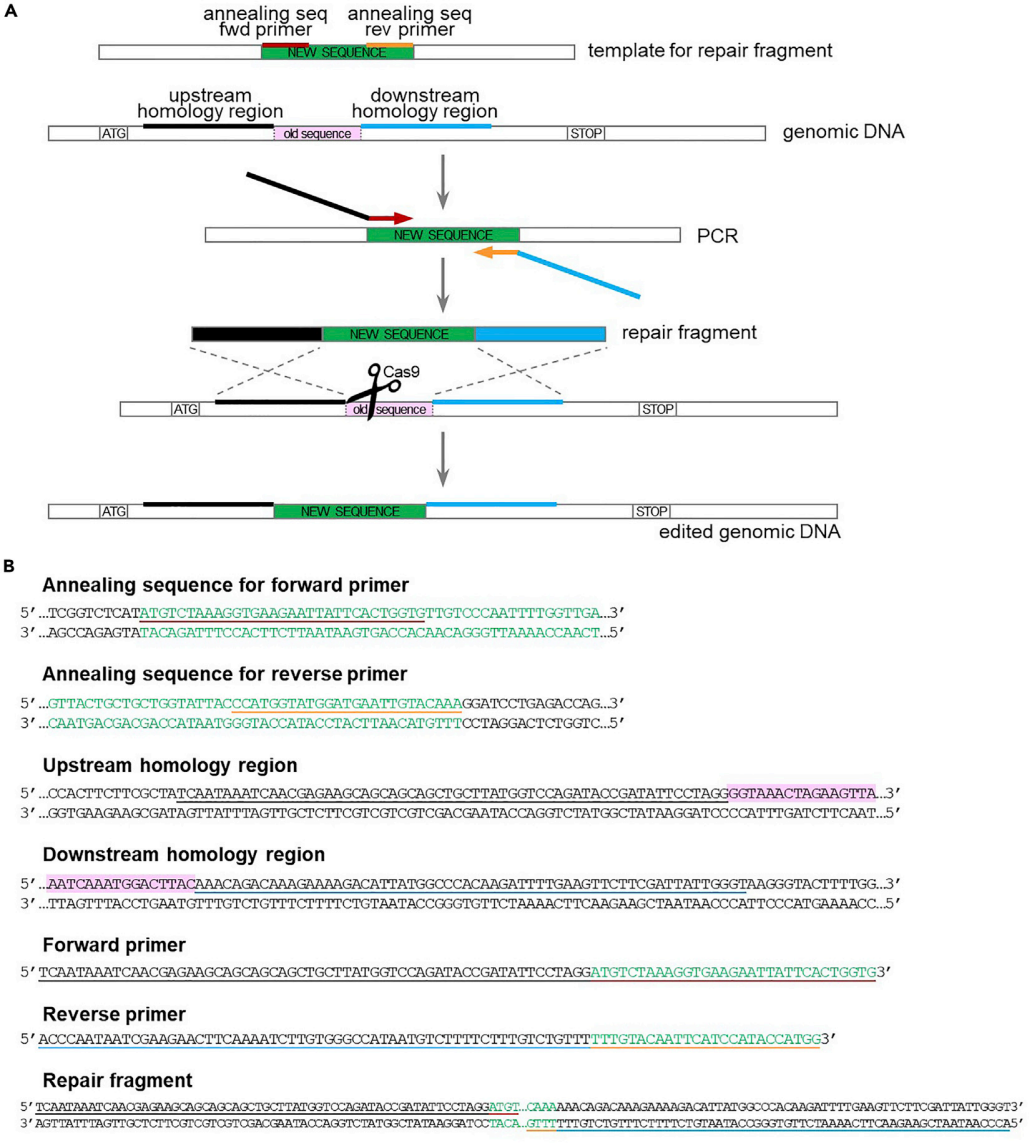
Design of repair fragment for sequence replacement (A) Schematic of the repair fragment design. The repair fragment is obtained through PCR amplification of the donor sequence (“new sequence”, in green) with primers annealing at the beginning (in dark red) and at the end (in orange) of the sequence, and carrying tails for the homology regions upstream (in black) and downstream (in blue) the sequence to be replaced (“old sequence”, in pink). After Cas9-dependent cleavage within the old sequence, repair fragment integration results in the insertion of the new sequence with concomitant deletion of the old sequence. (B) Example of primers design for sequence replacement (step 26). The old sequence is highlighted in pink. See main text for details.

It is possible to insert a donor sequence at any genomic location, with or without concomitant deletion of an endogenous sequence, based on the design of the repair fragment. The repair fragment for integration of the desired donor DNA sequence at a specific genomic position without deleting any endogenous sequence (assuming the sgRNA sequence contains the integration site) is made up of the 60 nucleotides upstream the integration site, followed by the donor sequence, followed by the 60 nucleotides downstream the integration site (Figure 7). The repair fragment for replacement of a specific genomic sequence with the desired donor sequence (assuming the sgRNA sequence is internal to the sequence to be replaced) is made up of the 60 nucleotides upstream the genomic sequence, followed by the donor sequence, followed by the 60 nucleotides downstream the genomic sequence (Figure 8). In both cases, the repair fragment is obtained by standard PCR amplification.

26. For both genome editing applications, design the primers for the repair fragment as follows.
  a. Design the annealing sequence for the forward primer on the top strand of the template for amplification of the donor sequence, according to the following rules:
    i. Start at the beginning of the donor sequence and extend the sequence in 5′ to 3′ direction.
    ii. The annealing sequence should be at least 20 nucleotides long.
    iii. If possible, the CG content should be between 35% and 65%.
    iv. The last nucleotide should be G or C, and the last 5 nucleotides should enclose at least 2 G/C.
    v. The Tm of the annealing sequence should be compatible with the Tm of the annealing sequence for the reverse primer (no more than 5°C difference).
  b. Design the annealing sequence for the reverse primer on the top strand of the template for amplification of the donor sequence, according to the following rules:
    i. Start at the end of the donor sequence and extend the sequence in 3′ to 5′ direction.
    ii. The annealing sequence should be at least 20 nucleotides long.
    iii. If possible, the CG content should be between 35% and 65%.
    iv. The last nucleotide should be G or C, and the last 5 nucleotides should enclose at least 2 G/C.
    v. The Tm of the annealing sequence should be compatible with the Tm of the annealing sequence for the forward primer (no more than 5°C difference).
  c. Design the homology regions for genomic integration.
    i. Upstream homology region for sequence integration: 60 nucleotides upstream the integration site.
    ii. Downstream homology region for sequence integration: 60 nucleotides downstream the integration site.
    iii. Upstream homology region for sequence replacement: 60 nucleotides upstream the sequence to be replaced.
    iv. Downstream homology region for sequence replacement: 60 nucleotides downstream the sequence to be replaced.
  d. Combine the annealing regions and the homology regions to create the primers.
    i. Forward primer: upstream homology region + annealing sequence for the forward primer.
    ii. Reverse primer: reverse complement downstream homology region + reverse complement annealing sequence for the reverse primer.
  e. Amplify the repair fragment through standard PCR.

***Note:*** Integration of the donor sequence at the desired genomic location or replacement of the desired genomic sequence with the donor sequence will disrupt the sgRNA sequence.

27. Proceed with yeast transformation (step 32).

### Design and production of the repair fragment for introduction of point mutations

**Timing: 2–3 h**

**Figure 9:**
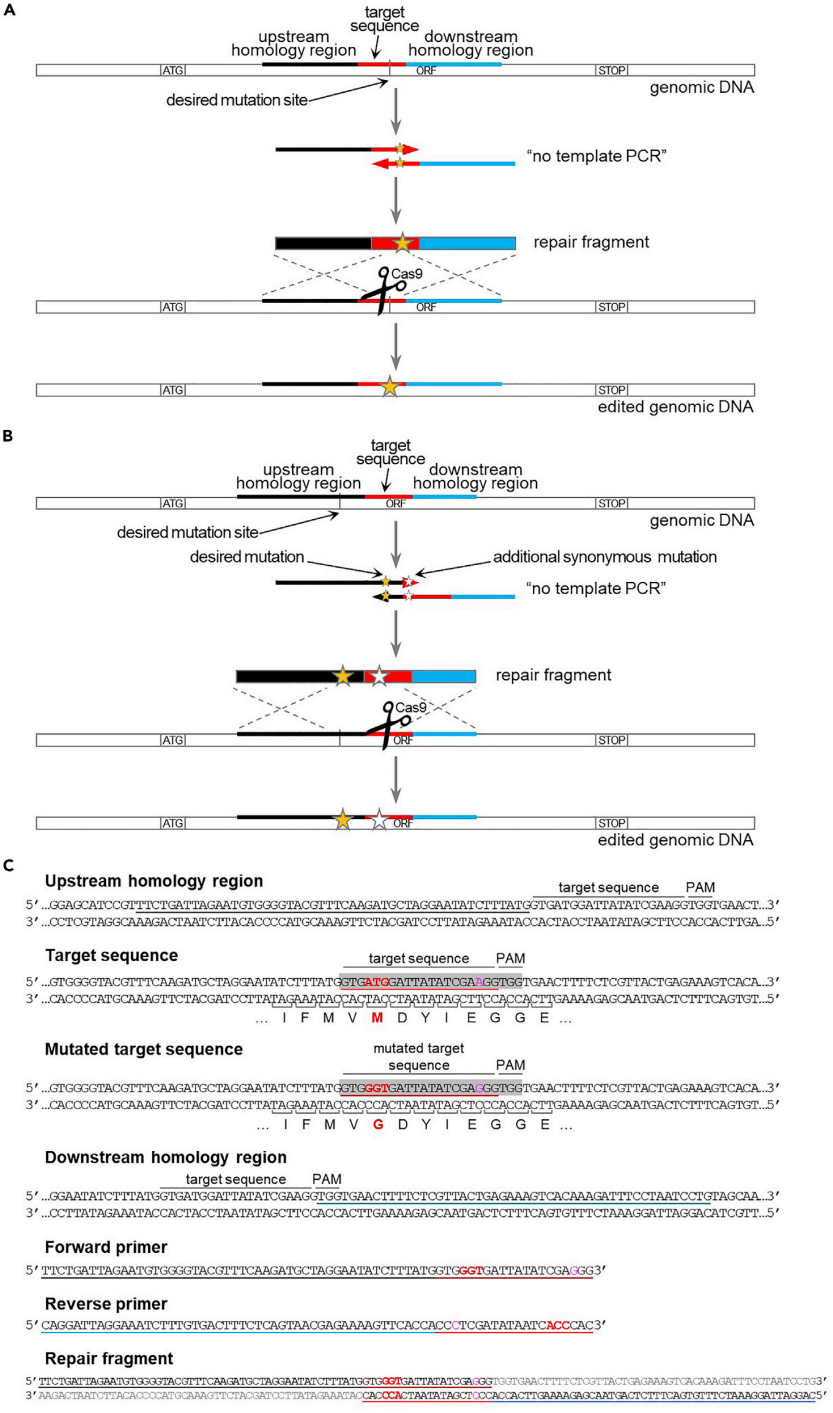
Design of repair fragment for the introduction of point mutations (A) Schematic of the repair fragment design when the desired mutation is located within the sgRNA sequence. The repair fragment is obtained through “no template PCR” amplification of partially overlapping primers containing the mutated target sequence and the homology regions upstream (in black) and downstream (in blue) the target sequence. The mutation is indicated by a yellow star. After Cas9-dependent cleavage near the mutation site, repair fragment integration results in the introduction of the desired mutation and the concomitant disruption of the target sequence. (B) Schematic of the repair fragment design when the desired mutation is located outside the sgRNA sequence. The repair fragment is obtained through “no template PCR” amplification of partially overlapping primers containing (part of) the target sequence and the homology regions upstream (in black) and downstream (in blue) the target sequence. The desired mutation (indicated by a yellow star) is introduced in the upstream or downstream homology region. Moreover, one or more additional synonymous mutations (indicated by a white star) are introduced in the target sequence. After Cas9-dependent cleavage near the mutation site, repair fragment integration results in the introduction of the desired mutation and the disruption of the target sequence. (C) Example of primers design for the introduction of point mutations, when the desired mutation is located within the target sequence (step 28). The sgRNA sequence is highlighted in gray. The mutated bases (ATG>GGT) and the corresponding amino acid change (M>G) are marked in red. The amino acid sequence is displayed using the one-letter code under the DNA sequence. Since the mutated bases are distal to the PAM, it is possible, out of precaution, to introduce an additional synonymous mutation (in magenta) in the target sequence near the PAM to ensure abrogation of Cas9 cleavage after repair. See main text for details.

If the sgRNA sequence includes the desired mutation site, the repair fragment for the introduction of point mutations is made up of the 50 nucleotides upstream the target sequence, followed by the 20-nucleotides target sequence, followed by the 50 nucleotides downstream the target sequence. The fragment carries the desired mutation either inside the target sequence or in the 50 nucleotides upstream or downstream. If needed, extra (synonymous) mutations are introduced to disrupt the sgRNA sequence. In case the sgRNA sequence does not include the desired mutation site, the repair fragment is designed similarly, but it is shifted a few nucleotides upstream or downstream, so that the mutation site and the target sequence are more or less equally distant from the edges of the repair fragment. In both instances, repair fragment is obtained by PCR amplification of partially overlapping primers (“no template PCR”, Figure 9).

28. In case the sgRNA sequence spans through the desired mutation site, design partially overlapping primers as follows.
  a. Forward primer: 50 nucleotides upstream target sequence + 20 nucleotides mutated target sequence.
  b. Reverse primer: reverse complement 50 nucleotides downstream target sequence + reverse complement 20 nucleotides mutated target sequence.
  c. The mutated sequence contains:
    i. The desired point mutation(s).
    ii. If necessary, one or multiple additional synonymous mutations in order to disrupt the sgRNA sequence, but still preserving the amino acid sequence.
  d. Introduction of additional synonymous mutations that disrupt the sgRNA sequence can be done in two ways:
    i. Mutate one of the “Gs” of the PAM site (NGG) so that the target sequence is no longer recognized (but avoid NAG, because it can still be recognized as a PAM).
    ii. Mutate at least 2 bases among the 10 PAM-proximal bases (this is enough to prevent binding of the sgRNA).

***Note:*** With such a design, the 20 3′-proximal nucleotides of the two primers anneal with each other, and each can provide the template for the extension of the other primer, yielding a complete 120 bp repair fragment.

29. In case the sgRNA sequence does not include the desired mutation site, design partially overlapping primers as follows.
  a. Select a 120-nucleotide sequence centered on the target sequence and the desired mutation site.
  b. Obtain the sequence of the repair fragment by adding the desired point mutation(s) and one or multiple additional synonymous mutations in order to disrupt the sgRNA sequence:
    i. Mutate one of the “Gs” of the PAM site (NGG) so that the target sequence is no longer recognized (but avoid NAG, because it can still be recognized as a PAM).
    ii. Alternatively, mutate at least 2 bases among the 10 PAM-proximal bases (this is enough to prevent binding of the sgRNA).
  c. Forward primer: the first 70 nucleotides of the repair fragment.
  d. Reverse primer: reverse complement of the last 70 nucleotides of the repair fragment.

***Note:*** With such a design, the 20 3′-proximal nucleotides of the two primers anneal with each other, and each can provide the template for the extension of the other primer, yielding a complete 120 bp repair fragment.

30. Amplify the repair fragment via “no template PCR”. Troubleshooting 3.
  a. Prepare the PCR mix:

**Table:** 

Component	Amount
5× Q5 buffer	40 μL
10 mM dNTPs	4 μL
10 μM forward primer	10 μL
10 μM reverse primer	10 μL
Q5 Polymerase	2 μL
H_2_O (Milli-Q)	134 μL
**Total**	**200 μL**
  b. Divide the PCR mix in 4 PCR tubes (50 μL per tube).
  c. Perform the PCR reaction in the thermocycler with the following program:

**Table:** 

PCR cycling conditions			
Steps	Temperature	Time	Cycles
Initial Denaturation	98°C	30 s	1
Denaturation	98°C	10 s	20 cycles
Annealing	53°C∗	30 s	
Extension	72°C	20 s	
Denaturation	98°C	10 s	10 cycles
Annealing + extension	72°C	20 s	
Final extension	72°C	5 min	1
Hold	4°C	forever	

∗ Annealing temperature of the overlapping region between the 2 primers (20 nucleotides). To calculate the annealing temperature, you can use the NEB Tm calculator tool (https://tmcalculator.neb.com).

    ***Note:*** The PCR program performs 20 cycles with an annealing temperature corresponding to the overlapping region (20 nucleotides), and then another 10 cycles with an annealing temperature = extension temperature (=72°C), corresponding to annealing of the whole primers (70 nucleotides).
  d. Pool the 4 PCR reactions together.
    ***Optional:*** Load 5 μL PCR product on agarose gel and verify PCR via DNA electrophoresis (expected band size: 120 bp).

31. Proceed with yeast transformation (step 32).

### Yeast transformation

**Timing: 6–7 days**

**Figure 10:**
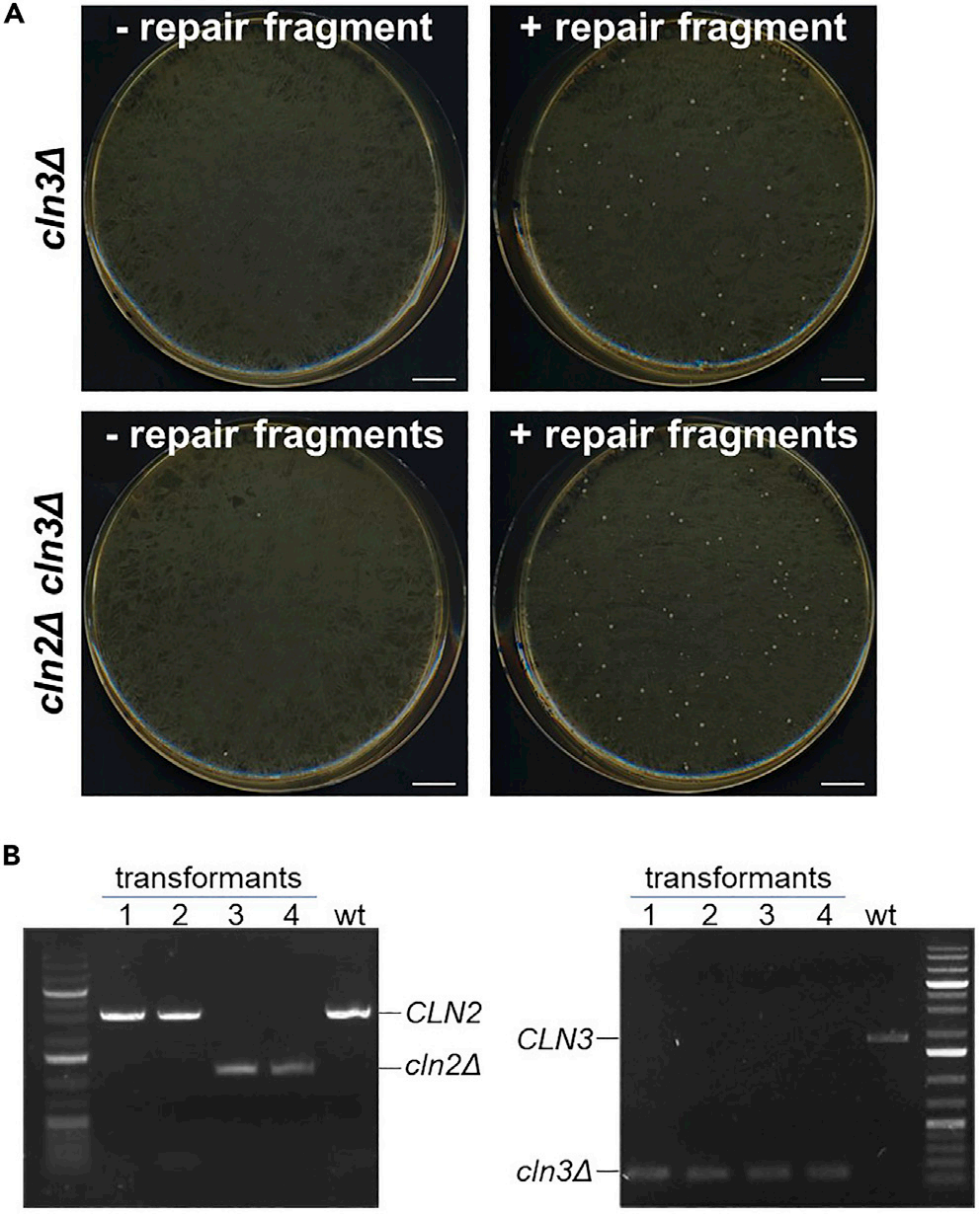
Examples of CRISPR transformation plates and verification of genome editing (A) Example of plates from yeast CRISPR transformation (step 33) for single (*cln3Δ*) and double (*cln2Δ cln3Δ*) gene knockout, with and without the repair fragment(s). Scale bar, 1 cm. (B) Example of gene editing verification via diagnostic PCR (steps 38 and 39). Four colonies from the double knockout transformation shown in panel A were PCR-tested for the deletion of genes *CLN2* and *CLN3*, respectively. In transformants 1 and 2 only *CLN3* gene was deleted, while deletion of *CLN2* gene failed. Conversely, transformants 3 and 4 display PCR products indicative of the simultaneous deletion of *CLN2* and *CLN3* genes.

The desired yeast strain is co-transformed with the Cas9+sgRNA(s) multi-gene plasmid and the appropriate repair fragment. As a negative control, the same transformation without the repair fragment is performed (Figure 10A).

***Note:*** The transformation protocol is adapted from the lithium acetate (LiAc)/SS carrier DNA/PEG method (Gietz and Schiestl, 2007).

32. Purify the repair fragment(s) with a PCR purification kit and measure DNA concentration with the NanoDrop (expected concentration: 50–250 ng/μL).

**Pause point:** Purified repair fragments can be stored at −20°C for several months.

33. Transform the desired yeast strain(s) using the LiAc method.
  a. Grow an overnight (12–20 h) yeast culture in 3 mL YPD liquid medium at 30°C, shaking at 300 rpm.
  b. The next morning, dilute cells 1:50 in 20 mL YPD liquid medium (sufficient for 6 transformations, if more transformations are needed increase the volume).
  c. Grow cells for 4–5 h at 30°C.
  d. Harvest the appropriate amount of cells.
    i. Measure the culture’s OD.
    ii. Dilute cells at OD 0.7.
    iii. Harvest 5 mL (∼ 10^8^ cells) cells for each transformation sample.
      ***Note:*** While harvesting the cells, calculate one extra sample (“N+1” rule). For example, for one genome editing transformation, 2 transformation samples are needed (with and without repair fragment), therefore 15 mL (for 3 samples) are harvested.
  e. Pellet cells in sterile 50 mL tubes at 2,300 g for 3 min at room temperature (RT, 20°C–25°C), discard the supernatant and resuspend cells in 25 mL sterile H_2_O.
  f. Pellet again at 2,300 g for 3 min at RT (20°C–25°C), discard the supernatant, resuspend cells in 1 mL 0.1 M LiAc and transfer in a sterile 2 mL Eppendorf tube.
  g. Spin cells at 5,000 g for 2 min at RT (20°C–25°C), discard the supernatant and resuspend cells in the appropriate amount of 0.1 M LiAc (80 μL per transformation sample), based on the number of cells harvested (“N+1” rule).
  h. Aliquot 100 μL cells for each transformation in “N” sterile 1.5 mL Eppendorf tubes.
  i. Spin cells at 5,000 g for 2 min at RT (20°C–25°C) and discard the supernatant.
  j. Add the Transformation mix (TMIX) components:

**Table:** 

Control mix (without repair fragment)	
Component	Amount
PEG (50% w/v in H_2_O)	240 μL
LiAc 1 M	36 μL
Carrier ssDNA (boiled 5 min and kept on ice)	25 μL
Cas9+sgRNA(s) plasmid	500 ng
ddH_2_O	up to 351 μL
**Total**	**351 μL**
    **Table:** 

CRISPR mix (with repair fragment)	
Component	Amount
PEG (50% w/v in H_2_O)	240 μL
LiAc 1 M	36 μL
Carrier ssDNA (boiled 5 min and kept on ice)	25 μL
Cas9+sgRNA(s) plasmid	500 ng
Repair fragment	3 μg
ddH_2_O	up to 351 μL
**Total**	**351 μL**

    ***Note:*** The total volume of DNA + ddH_2_O is 50 μL.
    **CRITICAL:** Pipette PEG slowly and carefully, since it is a very viscous solution.
    ***Note:*** If you want to perform simultaneous genome editing at two genomic loci with two sgRNAs and two repair fragments, 3 μg of each repair fragment should be added to the CRISPR mix. Furthermore, it is recommended to simultaneously perform also single locus genome editing (using each sgRNA and the corresponding repair fragment separately) as a control, to make sure that both sgRNAs work. Troubleshooting 6.
  k. Resuspend by vortexing and, if needed, pipetting up and down.
  l. Incubate at 42°C for 40 min (in a water bath).
  m. Pellet cells at 5,000 g for 2 min at RT (20°C–25°C) and carefully remove the TMIX (by pipetting).
  n. Resuspend cells in 1 mL YPD liquid medium and incubate 2–3 h at 30°C to allow expression of the resistance marker.
  o. Pellet cells at 5,000 g for 2 min at RT (20°C–25°C), resuspend in 150 μL sterile ddH_2_O and plate on the appropriate selective plates:
    i. YPD + G418 plates if the plasmid contains the KanR marker.
    ii. YPD + clonNAT plates if the plasmid contains the NatR marker.
    iii. YPD + HygB plates if the plasmid contains the HygR marker.
  p. Incubate plates at 30°C for 2–3 days.

34. Check the transformation plates to verify the proper functioning of the sgRNA(s): the plate with the repair fragment should have at least 10-fold more colonies than the plate without repair fragment (Figure 10A, see also expected outcomes and troubleshooting sections).
35. Choose 8–10 colonies from the transformation plate (with repair fragment!) and streak for single colony on selective plates (using the same selection drug used for the transformation).

**CRITICAL:** Streaking for single colonies is essential because the CRISPR genome editing event happens during the first cell divisions on plate, therefore colonies on the transformation plate might be heterogeneous.

**CRITICAL:** it is recommended to still keep the plasmid selection while streaking for single colony, to avoid false positives that lost the Cas9-gRNA plasmid but somehow are still alive on the transformation plate.

36. Make patches for temporary storage on YPD plates.
  a. For every re-streaked independent transformant, chose one individual colony and make a small patch on a YPD plate.
  b. If no single colony can be isolated for a particular transformant, that transformant should be left aside.

***Note:*** From this moment on, the selection for the plasmid marker is not needed anymore, and it is recommended to remove the selection to facilitate the loss of the plasmid (see removal of the Cas9+sgRNA(s) multi-gene plasmid). If your strain contains other antibiotic-resistance markers, you can decide to add one antibiotic to the YPD plates to minimize contamination.

37. Incubate the plate(s) at 30°C for 1 day.

***Optional:*** To save one day, you can use a small portion of the colony directly for genomic DNA extraction for colony PCR (see verification of genome editing), and patch the rest of the colony for temporary storage.

**Pause point:** plates can be stored at 4°C for a several days.

### Verification of genome editing

**Timing: 2–8 h**

Genomic DNA (gDNA) is prepared from the single colonies, and the desired genome editing event is verified by PCR and/or sequencing (Figure 10B).

38. Prepare gDNA from the isolated transformants with the LiAc-SDS method (Lõoke et al., 2011).
  a. For every transformant to be tested, prepare a 1.5 microcentrifuge tube with 100 μL of 200 mM LiAc, 1% SDS solution.
  b. With a pipette tip, take a tiny amount of cells from temporary storage patches (or directly from the single colonies).
  c. Resuspend cells in the LiAc-SDS solution.
  d. Incubate for 5 min at 70°C.
  e. Add 300 μL of 96%–100% ethanol and vortex.
  f. Spin down at 15,000 g for 3 min. Decant the supernatant.
  g. Wash pellet with 100 μL of 70% ethanol and vortex.
  h. Spin down at 15,000 g for 3 min. Remove ethanol as much as possible by pipetting.
  i. Place the open tubes near the flame or under the fume hood for 5–10 min to dry the residual ethanol.
  j. Dissolve the pellet in 100 μL of 5 mM Tris/HCl, pH 8.5, by incubating 10 min at 50°C.
  k. Spin down cell debris for 30 s at 15,000 g and transfer 80 μL of the supernatant in a new microcentrifuge tube.
  l. Use 0.5 μL for PCR (20 μL reaction volume).
39. In case of gene deletion, N- or C-terminal tagging, sequence integration, or sequence replacement, perform diagnostic PCR to verify the desired genome editing event.
  a. Prepare the PCR mix for “n + 2” samples, where n is the number of transformants to be tested. PCR mix for 1 sample:

**Table:** 

Component	Amount
5× Q5 buffer	4 μL
10 mM dNTPs	0.4 μL
10 μM forward primer	1 μL
10 μM reverse primer	1 μL
Q5 Polymerase	0.2 μL
H_2_O (Milli-Q)	12.9 μL
**Total**	**19.5 μL**
  b. For every test sample, mix 19.5 μL PCR mix + 0.5 μL gDNA.
    ***Note:*** Include a negative control from a non-transformed strain, and (if available) a positive control with the desired genome editing event.
  c. Perform the PCR reaction in the thermocycler with the following program:

**Table:** 

PCR cycling conditions			
Steps	Temperature	Time	Cycles
Initial Denaturation	98°C	30 s	1
Denaturation	98°C	10 s	35 cycles
Annealing	61°C∗	30 s	
Extension	72°C	30 s∗∗	
Final extension	72°C	5 min	1
Hold	4°C	forever	

∗ to calculate the annealing temperature of your primers, you can use the NEB Tm calculator tool (https://tmcalculator.neb.com).

∗∗ 20–30 s/Kb.
  d. Add 4 μL of 6× loading dye to each sample and load 20 μL on agarose gel. Analyze fragments via DNA electrophoresis. Troubleshooting 5.

40. In case of point mutations, perform PCR to amplify a fragment containing the mutation site.
  a. Prepare the PCR mix:

**Table:** 

Component	Amount
5× Q5 buffer	10 μL
10 mM dNTPs	1 μL
10 μM forward primer	2.5 μL
10 μM reverse primer	2.5 μL
gDNA	1 μL
Q5 Polymerase	0.5 μL
H_2_O (Milli-Q)	32.5 μL
**Total**	**50 μL**
  b. Perform the PCR reaction in the thermocycler with the following program:

**Table:** 

PCR cycling conditions			
Steps	Temperature	Time	Cycles
Initial Denaturation	98°C	30 s	1
Denaturation	98°C	10 s	35 cycles
Annealing	63°C∗	30 s	
Extension	72°C	30 s∗∗	
Final extension	72°C	5 min	1
Hold	4°C	forever	

∗ to calculate the annealing temperature of your primers, you can use the NEB Tm calculator tool (https://tmcalculator.neb.com).

∗∗ 20–30 s/Kb.

    ***Optional:*** Load 5 μL PCR product on agarose gel and verify PCR via DNA electrophoresis.
  c. Purify the PCR fragment with the PCR purification kit.
  d. Measure DNA concentration with the NanoDrop.
  e. Verify the presence of the desired mutation via Sanger sequencing, using one of the primers used for PCR amplification. Troubleshooting 5.

41. Select one or two verified transformants and proceed with plasmid removal.

***Note:*** It is possible to perform another round of CRISPR genome editing before plasmid removal, provided the second Cas9+sgRNA(s) multi-gene plasmid has a different selectable marker. The first plasmid will be likely lost in the process. At the end of the second round, loss of both plasmids has to be verified.

### Removal of the Cas9+sgRNA(s) multigene plasmid

**Timing: 5 days**

**Figure 11:**
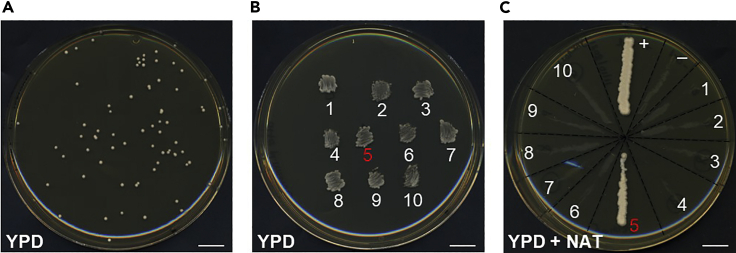
Plasmid removal after genome editing (A) Example of colonies after plating on non-selective plates for plasmid removal (steps 42–44). (B) Temporary storage patches on non-selective plates (step 45). (C) Verification of plasmid loss on selective plates (steps 45 and 46). As a consequence of plasmid loss, cells are unable to grow on the selective plate (in this example YPD+NAT). “+” and “-” indicate the positive and negative growth controls, respectively. In the example shown, all tested colonies have lost the plasmid, except colony 5. Scale bar, 1 cm.

Yeast cells lose the low-copy Cas9+sgRNA(s) multi-gene plasmid easily in the absence of selection. Verified strains are grown on non-selective medium and loss of the Cas9+sgRNA(s) multi-gene plasmid is verified via inability to grow on selective plates (Figure 11).

42. Inoculate a tiny amount of cells from the temporary storage patches of verified transformants in 3 mL YPD medium.
43. Grow an overnight (12–20 h) culture at 30°C, shaking at 300 rpm.
44. Plate the culture on (non-selective) YPD plates.
  a. Dilute the cultures in order to achieve ∼100 colonies on plate.
    i. Prepare a first dilution in a sterile 2 mL microcentrifuge tube: 70 μL cells + 930 μL ddH_2_O (sterile).
    ii. From the first dilution, perform two 1:100 serial dilutions in sterile 2 mL microcentrifuge tubes: 10 μL cells + 990 μL ddH_2_O (sterile).
  b. Plate 100 μL of the last dilution on YPD plates.
    ***Note:*** To minimize plate contamination, you can add the same antibiotic you added to the plates for temporary storage patches (step 36).
    ***Note:*** If the transformed yeast strain has growth defects, it may be necessary to adjust the growth time of the liquid culture, and/or the serial dilutions factor, in order to achieve ∼100 colonies on plate.
  c. Incubate plates at 30°C for 2 days.
45. Make new temporary storage patches and test plasmid loss.
  a. Patch 4 colonies per strain on YPD plates for temporary storage (it is again possible to add the antibiotic you added to the plates for temporary storage patches in step 36).
  b. At the same time (using the same inoculation loop or flat toothpick you used to make the patch), streak cells onto a selective plate for the Cas9+sgRNA(s) multi-gene plasmid (Figure 11C).
    ***Note:*** Include a positive and a negative control on selective plates.
  c. Incubate plates at 30°C for 1 day.
46. Verify plasmid loss: strains that have lost the plasmid do not grow on selective plates.
47. For each independent transformant (selected in step 41), freeze down (from the last temporary storage patches made in step 45) one colony that has lost the Cas9+sgRNA(s) multi-gene plasmid.

## Expected outcomes

This protocol consists of two main parts: i) cloning of Cas9 and the sgRNA(s) in a yeast expression vector via three subsequence Golden Gate assembly reactions and ii) yeast co-transformation with the Cas9+sgRNA(s) multi-gene plasmid and the appropriate repair fragment for genome editing.

An example of plates with *E. coli* colonies resulting from the three Golden Gate assembly reactions is shown in Figure 3A. The number of colonies on plate as well as the white/green colonies ratio may vary due to the transformation efficiency of the competent cells used and the efficiency of the different Golden Gate assembly reactions. However, in our hands the cloning procedure works very consistently and we were always able to isolate a correct Cas9+sgRNA(s) multi-gene plasmid at the end of the cloning workflow.

CRISPR/Cas9 technology allows precise, marker-free genome editing, provided that a suitable sgRNA and the proper repair fragment are employed. Figure 10 displays examples of successful yeast genome editing after transformation. The control transformation without the repair fragment is essential to assess the Cas9 cleavage efficiency associated with a specific sgRNA. An unrepaired double-strand break (DSB) in yeast is lethal. DSBs in yeast are efficiently repaired via homologous recombination (HR), provided a suitable homology template is available. In the absence of a donor template, repair can occur via the less efficient non-homologous end joining (NHEJ) pathway. If perfect NHEJ occurs, the repaired sequence remains identical (and can be cleaved again by Cas9), while imperfect NHEJ results in the presence of mutations in the proximity of the cleavage site. If the sgRNA works, very few colonies are expected in the control plate (resulting from imperfect NHEJ repair with mutation of the sgRNA sequence), while many more colonies (more than 10-fold) should appear in the plate with the repair fragment (since HR is way more efficient). Conversely, if both plates display a similarly high number of colonies, this is an indication that the selected sgRNA probably does not work well *in vivo* (see troubleshooting section).

If the sgRNA works, usually it is enough to test ∼10 colonies to identify at least one transformant bearing the desired genome editing event. The efficiency of genome editing depends on several factors: 1) the size of the deleted fragment: increased length = decreased efficiency; 2) the length of the homology regions at the ends of the repair fragment: increased length (up to 500 bp) = increased efficiency; 3) the distance between the genome editing site and the target sequence: increased distance = decreased efficiency.

Please consult the troubleshooting section for suggestions on how to improve genome editing efficiency.

## Limitations

The main limitation of CRISPR/Cas9 genome editing technology is the need for a suitable sgRNA sequence in the proximity of the editing site. If no suitable sgRNA is available, a different genome editing technique should be used, e.g., the *delitto perfetto* (Stuckey and Storici, 2013).

Secondly, the pre-assembled vectors we provide are suitable for cloning up to 2 sgRNAs together with Cas9, and contain only drug resistance yeast markers (KanR, NatR, HygR). If cloning of more than 2 sgRNAs in one vector and/or auxotrophic yeast marker are preferred, users should assemble the yeast expression vector(s) themselves (the MoClo-Yeast Toolkit design allows cloning of up to 4 sgRNAs in a single plasmid, and provides three auxotrophic markers) (Lee et al., 2015).

Another limiting factor of CRISPR/Cas9 genome editing is time. The whole procedure (from design of the sgRNA till genome editing verification and plasmid removal) takes at least three weeks. More specifically, the sgRNA cloning part can take a whole week. However, since no PCR amplification steps are required in the sgRNA cloning phase, the chance of introducing undesired mutations during sgRNA cloning is virtually absent. Furthermore, simultaneous expression of the Cas9 protein and the sgRNA from the same (low copy) plasmid circumvents the need for a host yeast strain with Cas9 stably integrated in the genome. All in all, we believe that the robustness and reliability of this protocol compensates well for its length.

## Troubleshooting

### Problem 1

It is difficult to distinguish white and green *E. coli* colonies obtained during sgRNA cloning (steps 7, 11, and 15).

### Potential solutions

• Incubation of the plates at 4°C for 5–8 h usually improves the detection of white versus green colonies.
• Alternatively, the difference between white and green can be better visualized by illuminating the plates with a blue or UV lamp.

### Problem 2

*E. coli* transformation with the Golden Gate assembly product yields only green colonies (steps 7, 11, and 15). This is an indication that probably the Golden Gate assembly reaction did not work.

### Potential solutions

• Double-check the design of the oligos for the sgRNA (step 5 of the before you begin section).
• Double-check the Golden Gate reaction table (especially the name of the plasmids and the plasmid concentrations).
• Repeat the Golden Gate reaction and *E. coli* transformation.

### Problem 3

Low yield of repair fragment obtained by “no template PCR” (steps 18 and 30).

### Potential solutions

Use the product of the “no template PCR” as a template for a standard PCR reaction, using two new 20-nucleotide primers annealing at the edges of the repair fragment.

### Problem 4

After CRISPR transformation there is no difference in the number of colonies in the presence or absence of the repair fragment (step 34). This is likely due to an insufficient amount of the repair fragment (if the number of colonies on both plates is very low), or to poor Cas9 cleavage because the sgRNA does not work (if many colonies grow on both plates).

### Potential solutions

• Increase the amount of the repair fragment (5–10 μg).
• Try a different sgRNA.

### Problem 5

The sgRNA works (clear difference −/+ repair fragment), but no clones carrying the desired editing event are detected after diagnostic PCR and/or sequencing (steps 38–40). This is an indication of low CRISPR efficiency.

### Potential solutions

To increase the efficiency of CRISPR genome editing, several strategies can be tried:

• Verify if there is any homologous sequence in the yeast genome that might be used as a donor sequence instead of the desired repair fragment. If this is the case, increase the amount of repair fragment (5–10 μg) and test many transformants (>20).
• Increase the length of the homology regions at the edges of the repair fragment to improve recombination efficiency (80–100 nucleotides).
• In case of gene knockout (or, more in general, deletion of any sequence), genome editing efficiency is inversely related to the length of the sequence to be deleted. To improve efficiency, increase the amount of repair fragment (5–10 μg) and test many transformants (>20).
• In case of large deletions (>5 Kb), to improve efficiency we suggest the simultaneous use of two sgRNAs, guiding Cas9 to cut near both ends of the sequence to be deleted, to facilitate loss of the intervening sequence. Also increasing the length of the repair fragment could contribute to improve efficiency.
• In case of point mutations, the distance between the target sequence and the desired mutation site affects the editing efficiency (increased distance = decreased efficiency). To improve efficiency, increase the amount of repair fragment (5–10 μg) and test many transformants (>20).

### Problem 6

Simultaneous genome editing with two (or more) sgRNAs does not work (no double-edited transformants are retrieved).

### Potential solutions

If the sgRNAs have been individually validated, two strategies are possible:

• Increase the amount of repair fragment the amount of transformants tested.
• Perform the two (or more) genome editing events sequentially in two independent transformations. If the Cas9+sgRNA multi-gene plasmids carry the same selection marker, removal of the plasmid is needed before the second transformation, otherwise both can be removed at the end of the editing procedure.

If the sgRNAs have not been individually validated, perform the CRISPR transformations with each sgRNA individually to verify them. The transformants of one of the transformations can be used as a starting strain to introduce the second genome editing event, as suggested above. If one of the sgRNAs does not work, try a different sgRNA.

## Resource availability

### Lead contact

Further information and requests for resources and reagents should be directed to and will be fulfilled by the lead contact, Andreas Milias-Argeitis (a.milias.argeitis@rug.nl).

### Materials availability

Plasmids generated in this study have been deposited to Addgene: pYTK-DN1 (#180282), pYTK-DN2 (#180283), pYTK-DN3 (#180284), pYTK-DN4 (#180285), pYTK-DN5 (#180286), and pYTK-DN6 (#180287).

## Data Availability

This study did not generate any datasets or code.

## Supplemental information

Data S1. CRISPR-Cas9 toolbox MoClo Assembly Excel spreadsheet, related to steps 4, 8, and 12
